# Stepwise reduction of an asymmetric π-expanded pyracylene towards the crystalline radical trianion†

**DOI:** 10.1039/d4sc08255a

**Published:** 2025-01-13

**Authors:** Yikun Zhu, Jan Borstelmann, Christian Neiss, Zheng Wei, Andreas Görling, Milan Kivala, Marina A. Petrukhina

**Affiliations:** a Department of Chemistry, University at Albany, State University of New York Albany New York 12222 USA mpetrukhina@albany.edu; b Organisch-Chemisches Institut, Universität Heidelberg Im Neuenheimer Feld 270 69120 Heidelberg Germany milan.kivala@oci.uni-heidelberg.de; c Lehrstuhl für Theoretische Chemie, Friedrich-Alexander Universität Erlangen-Nürnberg (FAU) Egerlandstraße 3 91058 Erlangen Germany; d Erlangen National High Performance Computing Center (NHR@FAU) Martensstr. 1 91058 Erlangen Germany

## Abstract

The chemical reduction of a pyracylene-hexa-*peri*-hexabenzocoronene-(HBC)-fused nanographene TPP was investigated with K and Rb metals to reveal its multi-electron acceptor abilities. The *in situ* reaction of TPP with the above alkali metals, monitored by UV-vis-NIR and ^1^H NMR spectroscopy, evidenced the stepwise reduction process. The use of different solvents and secondary ligands enabled isolation of single crystals of three different reduced states of TPP with 1, 2, and 3 electrons added to its π-system. This provided a unique set of carbanions with gradually increasing negative charge for in-depth structural analysis of the outcomes of controlled electron addition to a non-planar and asymmetric nanographene, using X-ray crystallographic, spectroscopic, and theoretical tools. EPR spectroscopy measurements of the mono- and triply-reduced TPP products revealed distinct EPR splitting patterns. DFT calculations demonstrated a notable difference in the spin density distribution of these two open-shell products and provided insights into experimental EPR data. Moreover, the influence of the counterions on the “naked” TPP anions was illustrated computationally.

## Abstract 2

The stepwise reduction by 1–3 electrons of a π-expanded pyracylene with K and Rb reveals that in-build core asymmetry affects the spin-density distribution in the mono- and trianion radicals, as confirmed by EPR spectroscopy and DFT calculations.

## Graphical abstract

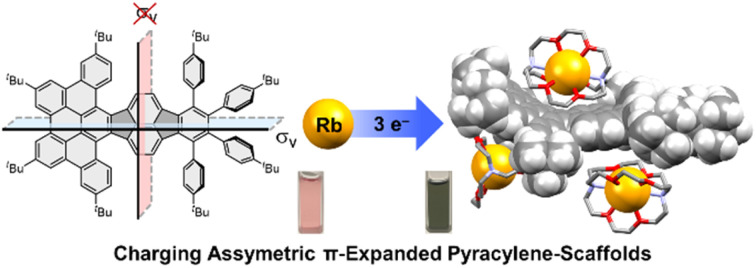

## Introduction

Intercalation of polycyclic aromatic hydrocarbons (PAHs) with alkali metals is becoming increasingly relevant for potential energy storage applications.^1^ In this context, assessment of electron accepting properties of various PAHs and studies of their reduced products in the different reduction states have been reported.^2,3^ Among them, trianions are rarely observed, however, they are particularly intriguing, due to their unique electronic structures. Trianions usually require higher symmetry elements, inducing nearly degenerate lowest unoccupied molecular orbitals (LUMOs), thereby stabilizing the negative charge. Therefore, thus far stable trianions have mostly been observed for *C*_3_-symmetric molecules, such as sumanene,^4^ truxene,^5^ decacyclene,^6^ or the *D*_6h_-symmetric hexa-*peri*-hexabenzocoronene (HBC).^7^ The triply-reduced products have also been reported for *C*_5v_-symmetric corannulene and its π-expanded derivative.^8,9^ These species often exhibit an open-shell character accompanied by Jahn–Teller effects.^10^ Consequently, not many examples of triply-reduced PAHs with lower symmetry have been observed thus far, which motivates for in-depth investigations of the symmetry influence.^11^

Pyracylene, formally a cut-out of the fullerene C_60_, has recently gained attention, due to its ability to reversibly accept two electrons.^12^ π-expansion of pyracylene has emerged as a particularly efficient strategy to induce curvature into the otherwise planar parent scaffold, which furthermore improves its electron accepting properties.^13^ Until recently, only a few examples of such π-expanded pyracylenes have been reported.^14^

**Fig. 1:**
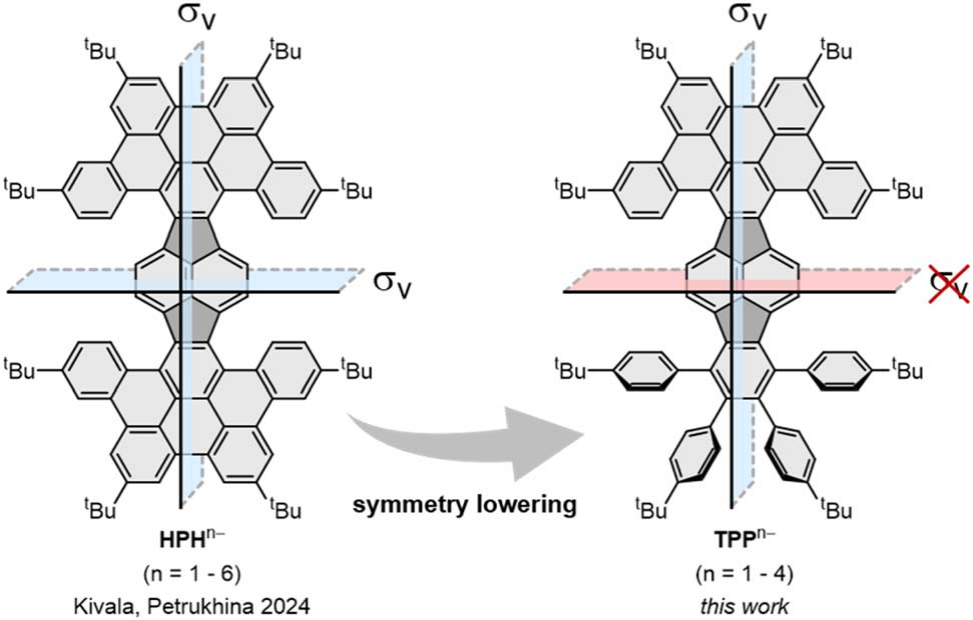
π-expanded pyracylenes as potent electron acceptors: symmetric HPH can be reduced up to the hexaanion, whereas lowering of the symmetry in TPP leads to formation of anions up to the tetraanion.^15,17^

In 2022, we reported the first systematic study of pyracylene-derived scaffolds, investigating the effect of successive π-expansion upon going from uncyclized octaphenyl-substituted dibenzopyracylene, *via* the half-cyclized tetraphenyl-substituted dibenzopyracylene (TPP) to the fully cyclized HBC-pyracylene hybrid (HPH).^15,16^ With the continuously increasing π-expansion, the curvature becomes more pronounced, resulting in a twisted boat-shaped conformation of the TPP and HPH scaffolds. Compared to the fully cyclized HPH, which has two HBC wings on the central pyracylene core with a largely increased molecular curvature, the fusion of only one HBC moiety generates a shallower half boat-shaped conformation with the depth of 3.25 Å (*vs.* 3.79 Å in HPH) on one side of TPP, whereas the other side with four phenyl substituents, namely a fragment of hexaphenylbenzene (HPB), remains nearly planar (Fig. 1). Cyclic voltammetry measurements showed that the π-expansion of the parent pyracylene efficiently modulates its propensity for a reversible electron uptake.

**Scheme 1:**
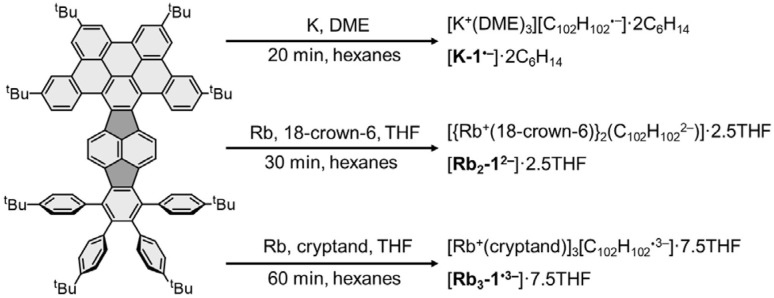
Chemical reduction of 1.

Previously, we explored the stepwise chemical reduction of HPH with alkali metals using single crystal X-ray diffraction, spectroscopic and theoretical tools and revealed its very high reduction limit up to the hexaanion and a unique boat-to-chair conformational change upon multi-electron addition.^17^ Herein, the stepwise chemical reduction of TPP featuring reduced symmetry (C_102_H_102_, 1, Scheme 1) was investigated with two Group 1 metals, K and Rb. The isolation of the gradually reduced products was targeted to reveal the structural and electronic consequences of adding multiple electrons to a more flexible nanographene with in-build core asymmetry.

## Results and discussion

### *In situ* spectroscopy investigations

**Fig. 2:**
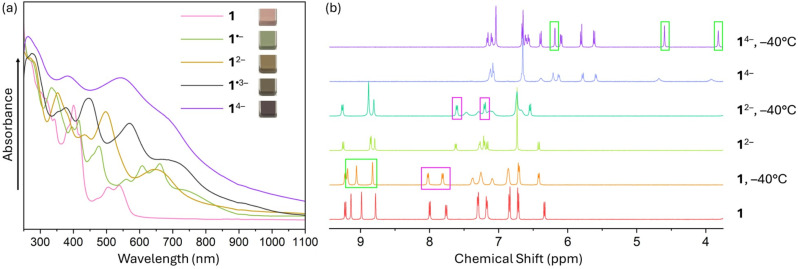
(a) UV-vis-NIR absorption spectra of TPP and its *in situ* generated anions by Rb metal in THF at 25 °C. (b) ^1^H NMR spectra of TPP and its *in situ* generated dianion and tetraanion by Rb metal in THF-*d*_8_ at 25 °C and −40 °C.

To probe the reduction limits, UV-vis-NIR absorption spectroscopy was used to monitor the stepwise reduction of TPP. The addition of Rb metal quickly changed the initial pink solution of neutral TPP to green (monoanion), brown (dianion), purple (trianion), and violet (tetraanion, Fig. 2a). Further confirmation of the four-fold reduction came from the *in situ* NMR spectroscopy measurements (Fig. 2b). Interestingly, the ^1^H chemical shifts of 1^2−^ in the NMR spectra remain largely in the similar range as the values of neutral 1, except for the two noticeably shifted protons (highlighted in pink boxes) on the pyracylene core. In contrast, the chemical shifts of 1^4−^ move to 3.8–7.2 ppm from 6.3–9.3 ppm in 1 and 1^2−^. The changes are especially pronounced for the protons of the HBC subunit, shifting from 8.8–9.2 ppm in 1 to 3.8–6.2 ppm (highlighted in green boxes) in 1^4−^. Importantly, the four-electron acquisition was proved by the ^1^H NMR spectroscopy to be fully reversible upon exposure to O_2_ (Fig. S5 and S6†).

### Single crystal X-ray diffraction analysis

**Fig. 3:**
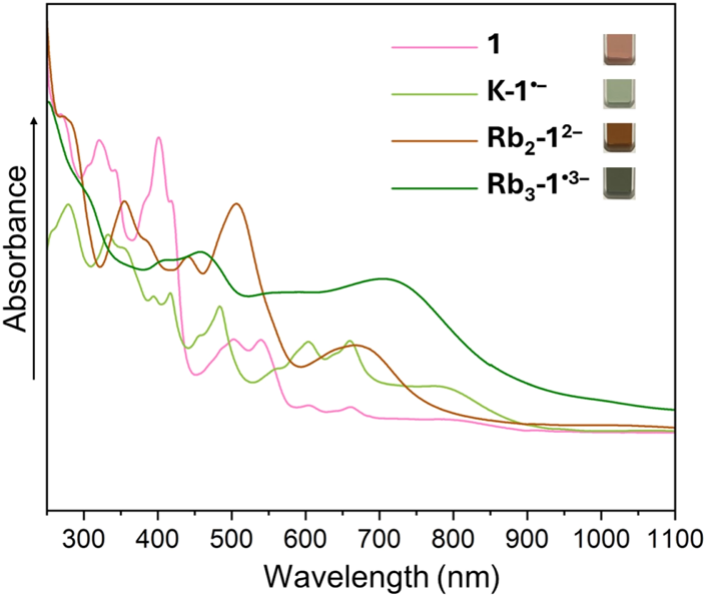
UV-vis-NIR absorption spectra of crystals of 1 and its reduced products dissolved in THF at 25 °C.

**Fig. 4:**
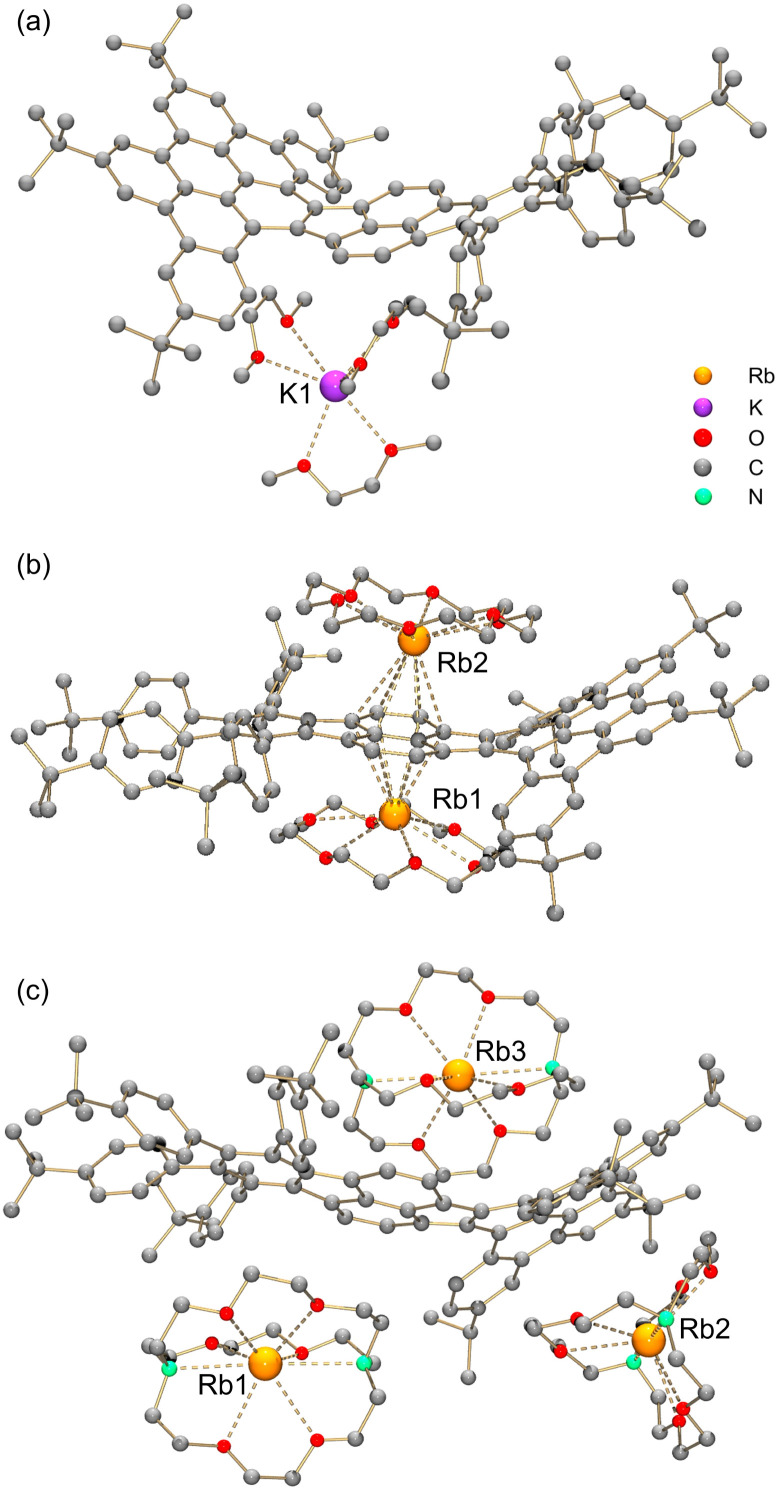
Crystal structures of (a) K-1˙^−^, (b) Rb_2_-1^2−^, and (c) Rb_3_-1˙^3−^, ball-and-stick model. Hydrogen atoms are omitted for clarity. The K⋯O distances range over 2.768(5)–2.818(9) Å. The Rb⋯O and Rb⋯N distances in Rb_3_-1˙^3−^ are 2.810(11)–2.963(11) Å and 3.003(14)–3.086(14) Å, respectively.^18^

By adjusting reaction conditions (Scheme 1), three different products with characteristic distinctive colors (Fig. 3) were prepared (see ESI for more details†). The controlled reduction with K metal in anhydrous 1,2-dimethoxyethane (DME), a strongly coordinating solvent, produced the grass-green solution of the TPP monoanion after 20 minutes. Slow diffusion of oxygen-/moisture-free hexanes into the reaction filtrate allowed the isolation of single crystals. The X-ray diffraction analysis revealed the formation of a solvent separated-ion product (SSIP) of the monoanion of 1 with the solvated K^+^ ion, [K^+^(DME)_3_][1˙^−^] (K-1˙^−^, Fig. 4a), crystallized with interstitial hexanes as [K-1˙^−^]·2C_6_H_14_. The use of Rb-metal and 18-crown-6 allowed the isolation of single crystals of the doubly-reduced product from the red brown THF solution. Its X-ray diffraction characterization confirmed the formation of a contact ion product (CIP), [{Rb^+^(18-crown-6)}_2_(1^2−^)]·2.5THF ([Rb_2_-1^2−^]·2.5THF, Fig. 4b). In contrast, the addition of [2.2.2]cryptand to the reaction mixture led to original color changes (Fig. S4†) compared to the use of Rb only (Fig. S1†). Specifically, the reaction mixture changed from pink to green (monoanion), red purple (dianion) and ended with brownish green color of the triply-reduced product (Fig. 3) after 60 minutes. Slow diffusion of anhydrous hexanes into the reaction filtrate allowed the isolation of a SSIP with three rubidium counterions, [Rb^+^(cryptand)]_3_[1˙^3−^] (Rb_3_-1˙^3−^, Fig. 4c), crystallized with multiple interstitial THF molecules as [Rb_3_-1˙^3−^]·7.5THF. Unfortunately, multiple attempts to isolate the tetra-reduced product of TPP were not successful, as the reactions always generated precipitates or thin needles, which did not provide sufficient diffraction for structure solution.

In two products, K-1˙^−^ and Rb_3_-1˙^3−^, the alkali metal ions fully wrapped by DME and [2.2.2]cryptand, respectively, remain solvent-separated from the corresponding anions. For reduced products with lower reduction states (−1 and −2), the lack of direct alkali metal coordination typically generates anions with higher symmetry.^19^ However, the isolation of the “naked” trianions is rare,^11^ so the influence of the cationic moieties on the highly reduced trianion core is unclear. In Rb_3_-1˙^3−^, the Rb2- and Rb3-moieties having multiple C–H⋯π interactions at different sides (convex *vs.* concave, Fig. S14†) of the HBC fragment and at the opposite sides of the *σ*_v_ plane (Fig. 1) could add additional distortion to the carbon backbone.

In contrast, the two Rb^+^-ions in Rb_2_-1^2−^ are bound to the same six-membered ring of the pyracylene core in an η^6^-fashion. One Rb^+^ ion nests inside the concave cavity of the core, while the other one coordinates from the opposite side (Fig. 4b). Both Rb^+^ ions are capped by 18-crown-6 molecules from the open end with the distances (2.842(5)–3.078(4) Å) comparable to the previously reported values.^20^ The intramolecular C–H⋯π interactions (2.376(5)–2.982(5) Å) between the 18-crown-6 ether and the concave side of the HBC unit force the Rb2 ion moving towards the HBC surface. This leads to a less symmetrical coordination pattern with the Rb⋯C contacts spanning over a larger range (3.155(2)–3.550(2) Å), in comparison to the Rb⋯C distances for Rb1 (3.230(2)–3.484(2) Å).

**Fig. 5:**
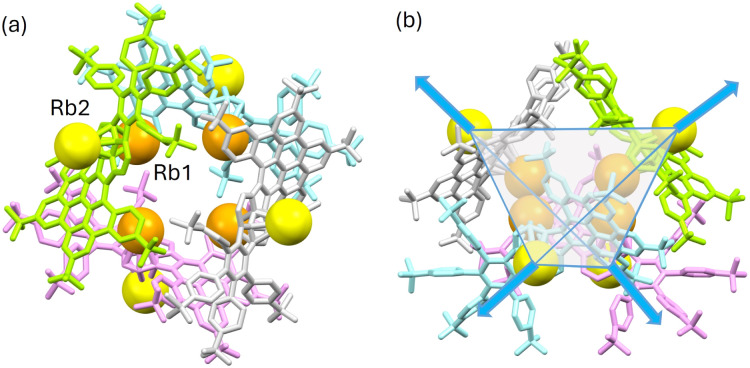
(a) Top view and (b) side view of the tetrahedral subunit [Rb_2_-1^2−^]_4_, mixed model. Anions, 1^2−^, are highlighted in different colors. Independent Rb^+^ cations are highlighted in different shades of orange. Hydrogen atoms and 18-crown-6 molecules are omitted.

In the solid-state structures of both SSIPs, the extended 1D columns are formed through C–H⋯π interactions between the cationic and anionic moieties (Fig. S15†). In contrast, in the solid-state structure of Rb_2_-1^2−^, multiple C–H⋯π interactions (2.603(2)–2.875(2) Å) between the Rb1-centered cation and the HBC surface of adjacent 1^2−^ contribute to the formation of a tetrahedral subunit consisting of four Rb_2_-1^2−^ molecules (Fig. 5a). In this subunit, four Rb1 cationic moieties are wrapped by the anions as an inner core, and the remaining four Rb2-based moieties stay at the four corners of the tetrahedron. An additional C–H⋯π interaction of 2.784(5) Å between the 18-crown-6 on Rb2 and the HBC unit from the adjacent subunit (along the blue arrows in Fig. 5b) further expands the solid-state structure into a 3D network (Fig. S16†).

### Charge-dependent core deformation

**Scheme 2:**
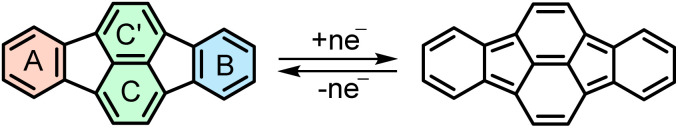
Structural evolution of the pyracylene core upon electron uptake from 1 towards 1˙^−^, 1^2−^, and 1˙^3−^. The single and double bonds on the right structure indicate the trends in bond length changes.

A comparison between the neutral parent and three reduced products reveals gradual C–C bond length changes upon electron addition, especially over the central pyracylene core (Scheme 2). From charges of 0 to −3, the C–C bond distances around the two five-membered rings of the pyracylene core experience a distinct bond length change: the edge bonds C2–C3 and C6–C9 become gradually shortened and reach the aromatic region (Table 1). At the same time, the C2–C2′ bonds are elongated from 1.431(3) Å to 1.441(5) Å in 1˙^−^, 1.473(3) Å in 1^2−^, and 1.485(9) Å in 1˙^3−^. The same bond distance change was reported previously upon controlled chemical reduction of HPH.^17^ For the six-membered rings (C/C′ in Scheme 2), the C4–C5 bond distances gradually decrease (Table 1, 1.369(9) Å in 1˙^3−^*vs.* 1.427(4) Å in 1) upon stepwise electron addition and become similar to the central C7–C8 bond. On the other hand, the bonds C3–C4 and C5–C6 are elongated, and the extent of changes is similar to those observed in the reduced HPH anions.^17^

**Table 1:** Selected C–C distances (Å) in 1, 1˙^−^, 1^2−^, and 1˙^3−^, along with a labelling scheme^a^

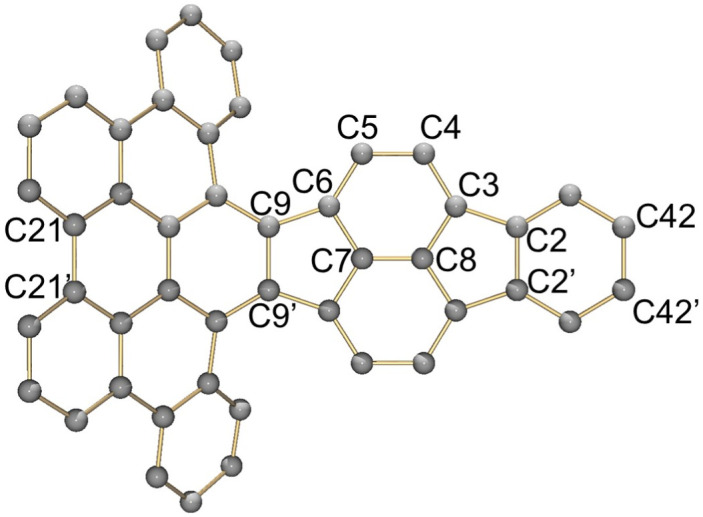				
	1	1˙^−^	1^2−^	1˙^3−^
C2–C2′	1.429(3)	1.433(5)	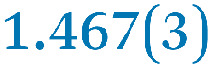	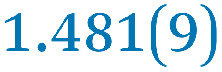
C2–C3	1.485(3)	1.471(3)	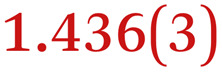	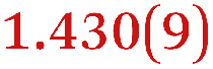
C3–C4	1.386(3)	1.408(3)	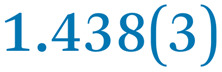	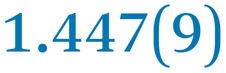
C3–C8	1.409(3)	1.415(3)	1.411(3)	1.433(9)
C4–C5	1.427(4)	1.406(3)	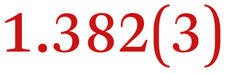	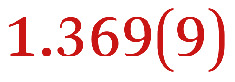
C5–C6	1.396(3)	1.417(3)	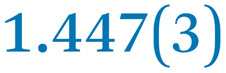	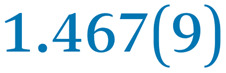
C6–C7	1.410(3)	1.417(3)	1.412(3)	1.418(8)
C7–C8	1.356(4)	1.374(4)	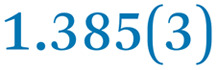	1.369(9)
C6–C9	1.491(3)	1.467(3)	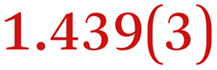	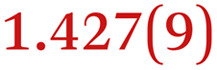
C9–C9′	1.433(3)	1.450(4)	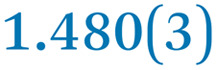	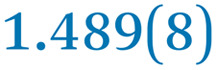
C21–C21′	1.473(3)	1.464(4)	1.459(3)	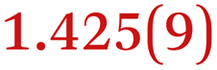
C42–C42′	1.409(3)	1.408(6)	1.420(3)	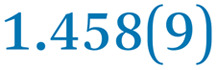

a The ^*t*^Bu-groups and σ-phenyl rings are omitted for clarity. The significantly elongated/shortened C–C bonds compared to 1 are highlighted in blue/red.

Lastly, the asymmetry of the core structure (HBC *vs.* HPB subunits) leads to different bond length responses of the two molecular halves: the C21–C21′ bond of the HBC core becomes significantly shorter (1.425(9) Å) than in the neutral parent (1.473(3) Å). In contrast, the C42–C42′ bond on the phenyl-substituted side is elongated up to 0.05 Å in 1˙^3−^, in comparison with neutral 1. Notably, the Rb-coordination in Rb_2_-1^2−^ does not influence the overall trends observed along the series (Table 1).

The stepwise chemical reduction also leads to the overall curvature change of the TPP anions. The two-fold reduction reduces the bowl depth, whereas the addition of the third electron increases the depth and curvature (Table S3†). The curvature changes in the crystal structures are probably due to crystal packing effects. Although TPP and its anions are supposed to have *C*_s_ symmetry, the ionic interactions make the two halves (left and right of the *σ*_v_ plane in Fig. 1) unsymmetric in the solid-state structures. This is especially pronounced in 1˙^3−^ (Fig. S17†), as revealed by the difference in helical and twist angles (Table S3†).

### EPR spectroscopic analysis of the radical anions

To better understand the effect of the asymmetric carbon framework on the properties of radical anions, especially the trianion, EPR spectroscopy was used to investigate the reduced radical products. Due to lack of hyperfine details in the EPR spectra of the solid crystalline samples (Fig. S7 and S8†), the *in situ* generated radical anions with different metal interactions were prepared and investigated (see ESI for more details†).

**Fig. 6:**
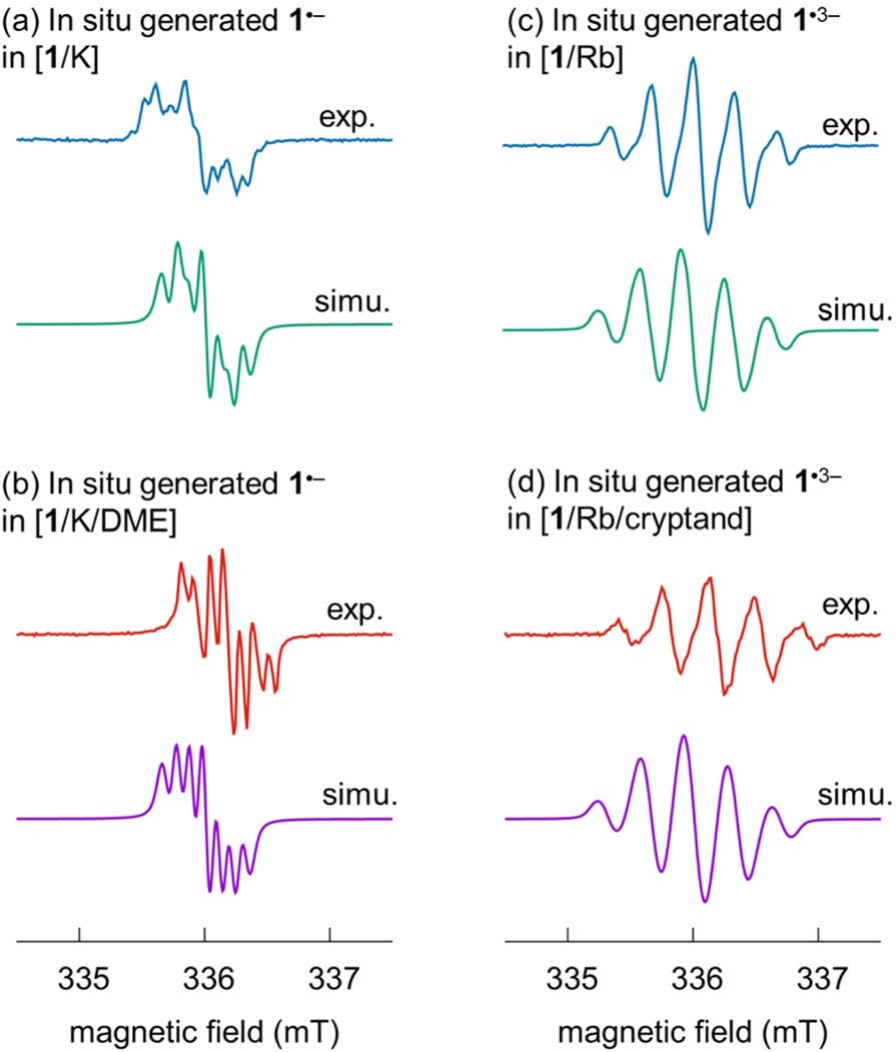
Experimental and simulated EPR spectra of (a and b) the monoanion 1˙^−^ and (c and d) trianion 1˙^3−^ in solution under different conditions. Experimental spectra were re-scaled individually to yield comparable intensities.

The spectra of the mono- and trianionic radicals clearly reveal different EPR patterns. For 1˙^−^, a set of multiplets are found for both *in situ* generated spectra (Fig. 6). Compared to the EPR spectrum of [1/K] in THF, which allows direct metal coordination and generates broader overlapping peaks, the EPR spectrum of the SSIP obtained from [1/K/DME] shows improved resolution with better peak separation. In the case of the trianion radical 1˙^3−^, an obviously broadened EPR pattern is observed with five peaks in the ratio of 1 : 3 : 5 : 3 : 1 and a hyperfine coupling constant of 0.330 mT (9.2 MHz) in both spectra obtained under different conditions ([1/Rb] *vs.* [1/Rb/cryptand], Fig. S8†). To provide further insights about the radical species, theoretical analysis was carried out.

## Theoretical analysis

To gain deeper insight into the electronic features of the differently charged states of 1, calculations on the density-functional theory (DFT) level were conducted (using the software package ORCA,^21^ see ESI for computational details†). In order to separate inherent properties of 1^*n*−^ from complexation effects simulations were carried out both without and with counterions present, *i.e.*, the [K^+^(DME)_3_][1˙^−^], [{Rb^+^(18-crown-6)}_2_(1^2−^)] and [Rb^+^(cryptand)]_3_[1˙^3−^] complexes were considered. In all cases, the structures were placed in a polarizable continuum mimicking THF (*ε*_r_ = 7.25).^22^

**Fig. 7:**
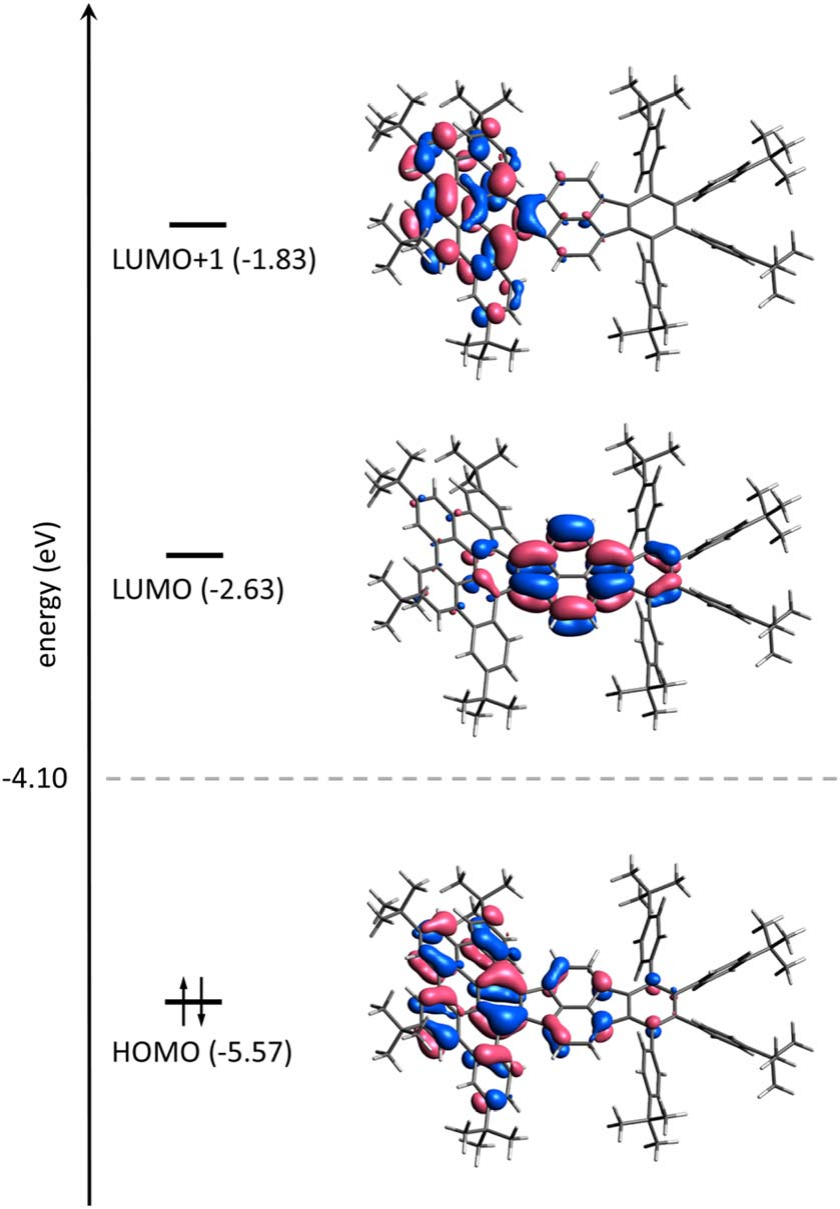
Frontier molecular orbital scheme of 1 on the DFT level (PBE0 level incl. PCM(THF), see ESI for details†). Lobes are plotted for an isovalue of 0.03 bohr^−(3/2)^. The Fermi energy is indicated by a dashed line; energy levels are not to scaled.

Fig. 7 shows the frontier molecular orbitals (MOs) of 1. In a simple picture, the reductions correspond to filling electrons in the lowest unoccupied MO (LUMO) and the next higher MO (LUMO+1). Indeed, the first two reduction steps of 1 are localized on the pyracylene core of 1 as can be seen from electron density difference maps (Fig. S18†) and closely resemble the ones of HPH recently investigated by us.^17^ The spin density of 1˙^−^, which forms a doublet state, confirms this as well (Fig. S19a†). The third reduction, however, involves essentially the HBC flank of the molecule (Fig. 7 and Fig. S18†), which is reflected by the calculated spin density of 1˙^3−^ (Fig. S19b†). In addition, the calculated MO gaps are in good agreement with the experimental UV-vis-NIR patterns. Stepwise reduction induces red-shifts of *λ*_max_ and increased absorbance intensities in the UV-vis-NIR spectra (Fig. 2a), indicating progressive reduction of the energy gaps upon sequential electron addition.

The resulting increase of (anti-) bonding between certain atom pairs leads to changes in bond length (Table S5†) that are broadly in agreement with the experimentally determined changes reported in Table 1. Considering the counterions explicitly does not influence the bond lengths significantly (Table S5†). However, the counterions lift the *σ*_v_ symmetry plane, which leads to some slightly different bond lengths in the two halves of the molecule (indicated in Table S5† by two values for a bond length). The largest effects occur for the dianion [{Rb^+^(18-crown-6)}_2_(1^2−^)] due to the direct Rb^+^ contact with the π-system.

The bond lengths also provide insight on the (anti)-aromaticity evolution of individual carbon rings upon reduction *via* the harmonic oscillator model of aromaticity (HOMA, Table S6†).^23^ Consistent with the Clar sextets shown in Scheme 2, the six-membered rings are strongly aromatic, while the five-membered rings are essentially non-aromatic (or slightly anti-aromatic) in neutral 1. Upon reduction, the aromatic character of the six-membered rings decreases, while the five-membered rings gain more aromatic character, driven by the formation of a cyclopentadienyl-like π-system. As the third reduction does hardly affect the pyracylene core, the HOMA values of the pyracylene rings are very similar between the dianion and trianion.

The spin densities (Fig. S19†) give rise to the measured EPR patterns. In the case of the monoanion, the four hydrogen atoms directly bound to the pyracylene core (Table S7†) dictate the splitting pattern in accordance with exhibiting the largest spin density in their vicinity; all other hyperfine coupling constants (HFC) are well below 0.5 MHz. Coordination of the counterion leads to small shifts of the HFC parameters. For the trianion, however, the spin density is essentially located on the HBC part of the molecule. The resulting EPR pattern is therefore dominated by the HFCs of six hydrogen atoms with the largest spin density on the HBC core (Table S7†). The largest HFC parameters and simulated EPR spectra are in very good agreement with the experimentally determined values (Fig. 6, see above). Because there are both larger and smaller HFC parameters (there are more hydrogen atoms close to the HBC moiety than to the pyracylene core) compared to the monoanion, the EPR spectrum of the trianion has a different (broader) shape than that of the monoanion. Therefore, the effect of the counterions is hardly visible in the EPR spectrum of the trianion. Both for the mono- and trianion, there is no significant spin density on the counterions.

## Conclusions

We report the stepwise chemical reduction of an asymmetric pyracylene-HBC fused nanographene with four lateral phenyl substituents (TPP). The *in situ* spectroscopic investigation of TPP reveals a multistep reduction process, which is fully reversible. Stopping the reaction at the mono-, doubly- and triply-reduced stages allowed the isolation of the corresponding anions with K- and Rb-based cationic counterparts, thus enabling the evaluation of the influence of sequential electron addition on the asymmetric polyarene. Moreover, the theoretical studies reveal that a simple MO picture of the neutral compound 1 can explain the observed bond length changes arising from the successive reduction. While the first two reductions clearly occur on the pyracylene core, where the LUMO is predominantly localized, the third reduction involves the cyclized hexabenzocoronene-like moiety and consequently the LUMO+1 of the molecule. This is also reflected in the measured EPR patterns, which are consistent with the differing spin densities of the mono- and trianion of 1 that lead to significantly different hyperfine couplings. Our findings suggest that the formation of trianion radicals is not exclusively limited to the reduction of highly symmetric PAHs. Asymmetric polycyclic scaffolds may also favor their formation, which opens new avenues in the molecular design of triply-reduced open-shell systems for both fundamental studies and potential charge and spin storage applications.

## Data availability

The data that support the findings of this study are provided in the ESI.†

## Author contributions

M. K. and M. A. P. conceived and supervised the project. J. B. synthesized the neutral compound 1. Y. Z. performed chemical reduction, crystallization, and all spectroscopic characterization of the stepwise reduced products. X-ray diffraction experiments and refinement were performed by Z. W. DFT calculations were performed and analysed by C. N. All authors contributed to the manuscript preparation and editing. Funding for this work was secured by M. K., A. G., and M. A. P.

## Conflicts of interest

There are no conflicts to declare.

## Supplementary Material

SC-016-D4SC08255A-s001

SC-016-D4SC08255A-s002
