# Supplementary material for: Stepwise reduction of an asymmetric π-expanded pyracylene towards the crystalline radical trianion
Source: Chem Sci. 2025 Jan 13;16(7):3211–7. doi: 10.1039/d4sc08255a (PMC11744371; doi:10.1039/d4sc08255a)
Supplement: SC-016-D4SC08255A-s001 [file SC-016-D4SC08255A-s001.pdf]

Supplementary Information (SI) for Chemical Science.
This journal is © The Royal Society of Chemistry 2025

Supporting Information

For

Stepwise Reduction of an Asymmetric π-Expanded Pyracylene Towards the Crystalline Radical Trianion

Yikun Zhu,[‡a] Jan Borstelmann,[‡b] Christian Neiss,[‡c] Zheng Wei,[a] Andreas Görling,[c,d] Milan Kivala,[*b] and Marina A. Petrukhina[*a]

a. Department of Chemistry, University at Albany, State University of New York, Albany, New York 12222, USA. E-mail: mpetrukhina@albany.edu

b. Organisch-Chemisches Institut, Universität Heidelberg, Im Neuenheimer Feld 270, 69120 Heidelberg, Germany. Email: milan.kivala@oci.uni-heidelberg.de

c. Lehrstuhl für Theoretische Chemie, Friedrich-Alexander Universität Erlangen-Nürnberg (FAU), Egerlandstraße 3, 91058 Erlangen, Germany

d. Erlangen National High Performance Computing Center (NHR@FAU), Martensstr. 1, 91058 Erlangen, Germany

I. Materials and Methods S1

II. UV-vis-NIR Spectroscopic Investigation S3

III. NMR Spectroscopic Investigation S7

IV. EPR Spectroscopic Investigation S9

V. Crystal Structure Solution and Refinement S10

VI. Computational Studies S20

VII. References S28

I. Materials and Methods

All manipulations and reaction procedures were carried out using break-and-seal and glove-box techniques under an atmosphere of argon (Airgas, 99.999%).[1] Tetrahydrofuran (THF) and hexanes (Sigma Aldrich) were dried over Na/benzophenone and distilled prior to use. 1,2-Dimethoxyethane (≥99%, DME) and tetrahydrofuran-d_8 (≥99.5 atom %D, Sigma Aldrich) was dried over NaK_2 alloy and vacuum-transferred. Potassium (98 %), rubidium (99.5 %), 18-crown-6 (99%), and [2.2.2]cryptand (99.0%) were purchased from Sigma Aldrich and used as received. $C_{102}H_{102}$ (**1**, **TPP**) was prepared according to the previously reported procedure.[2] UV-vis-NIR absorption measurements were carried out in a 1 cm path length quartz cuvette with PTFE cap at 298 K using a Jasco V770 spectrophotometer. The ^1H NMR spectra were recorded on a Bruker Ascend-500 spectrometer (500 MHz for ^1H). Chemical shifts (δ) are reported in parts per million (ppm) and referenced to the resonances of the corresponding solvent used. The EPR spectra were collected with an ADANI SPINSCAN X EPR spectrometer with the following parameters: center field = 336.00 mT, sweep width = 15 mT, sweep time = 60 s, modulation amplitude = 100 uT, attenuation = 20 dB, temperature = 30.7 °C. The extreme oxygen- and moisture sensitivity of all reduced products, along with the presence of interstitial hexane and THF molecules, prevented obtaining elemental analysis data.

$[K^+(DME)_3][C_{102}H_{102}^{\bullet-}]\cdot 2C_6H_{14}$ ($[K\text{-}1^{\bullet-}]\cdot 2C_6H_{14}$)

DME (1.0 mL) was added to a customized glass system containing excess K metal (3.0 mg, 0.077 mmol) and **TPP** (**1**, 3.0 mg, 0.002 mmol). The reaction mixture was stirred at 25 °C under argon for 20 minutes. The initial red color (neutral ligand) changed to green in 15 minutes. The mixture was filtered after 20 minutes, and the green filtrate was layered with 1.5 mL of anhydrous hexanes. The ampule was sealed under argon and stored at 5 °C. After 7 days, green plate-shaped crystals formed in the ampule. The ampule was opened inside the glovebox, and the remaining solution was removed. Crystals were washed with anhydrous hexanes and dried. Crystals were weighted inside the glovebox and the yield was calculated based on the amount of **TPP**. Yield: 3.0 mg, 73%. UV-vis-NIR (THF): λ_{max} 258 (sh), 279, 332, 351 (sh), 393, 417, 456 (sh), 484, 562 (sh), 602, 659, 775 (sh) nm.

$[Rb^+(18\text{-crown-6})]_2[C_{102}H_{102}{}^{2–}]·2.5C_4H_8O$ ($[Rb_2\text{-}1^{2–}]·2.5C_4H_8O$)

THF (1.0 mL) was added to a customized glass system containing excess Rb metal (3.0 mg, 0.035 mmol), 18-crown-6 (1.2 mg, 0.004 mmol), and **TPP** (**1**, 3.0 mg, 0.002 mmol). The reaction mixture was stirred at 25 °C under argon for 30 minutes. The initial red color (neutral ligand) changed to green in 10 minutes followed by purple-brown in 25 minutes. The mixture was filtered after 30 minutes, and the purple-brown filtrate was layered with 1.5 mL of anhydrous hexanes. The ampule was sealed under argon and stored at 5 °C. After 14 days, black plate-shaped crystals formed in the ampule. The ampule was opened inside the glovebox, and the remaining solution was removed. Crystals were washed with anhydrous hexanes and dried. Crystals were weighted inside the glovebox and the yield was calculated based on the amount of **TPP**. Yield: 1.5 mg, 33%. UV-vis-NIR (THF): λ_{max} 269, 354, 384 (sh), 440, 506, 666 nm.

$[Rb^+(\text{cryptand})]_3[C_{102}H_{102}{}^{•3–}]·7.5C_4H_8O$ ($[Rb_3\text{-}1^{•3–}]·7.5C_4H_8O$)

THF (1.0 mL) was added to a customized glass system containing excess Rb metal (3.0 mg, 0.035 mmol), [2.2.2]cryptand (2.5 mg, 0.006 mmol), and **TPP** (**1**, 3.0 mg, 0.002 mmol). The reaction mixture was stirred at 25 °C under argon for 60 minutes. The initial red color (neutral ligand) changed to green in 10 minutes, followed by purple in 25 minutes and brownish green in 45 minutes. The mixture was filtered after 60 minutes, and the brownish green filtrate was layered with 1.5 mL of anhydrous hexanes. The ampule was sealed under argon and stored at 5 °C. After 7 days, dark green block-shaped crystals formed in the ampule. The ampule was opened inside the glovebox, and the remaining solution was removed. Crystals were washed with anhydrous hexanes and dried. Crystals were weighted inside the glovebox and the yield was calculated based on the amount of **TPP**. Yield: 3.0 mg, 41%. UV-vis-NIR (THF): λ_{max} 441, 454, 562 (sh), 705 nm.

II. UV-vis-NIR Spectroscopic Investigation

Sample preparation: THF (3.0 mL) was added to a quartz cuvette containing **1** (0.2 mg, 0.0001 mmol) inside a glovebox. The cuvette was closed tightly with a PTFE cap with a piece of Rb metal (1.0 mg, 0.0117 mmol) attached to the inner surface, which prevented the initial reaction and allowed control over the reduction process. The capped cuvette was wrapped with Parafilm and removed from the glovebox. The UV-vis-NIR absorption spectra were measured without Rb metal initially and then monitored with Rb metal at different reaction times at 25 °C.

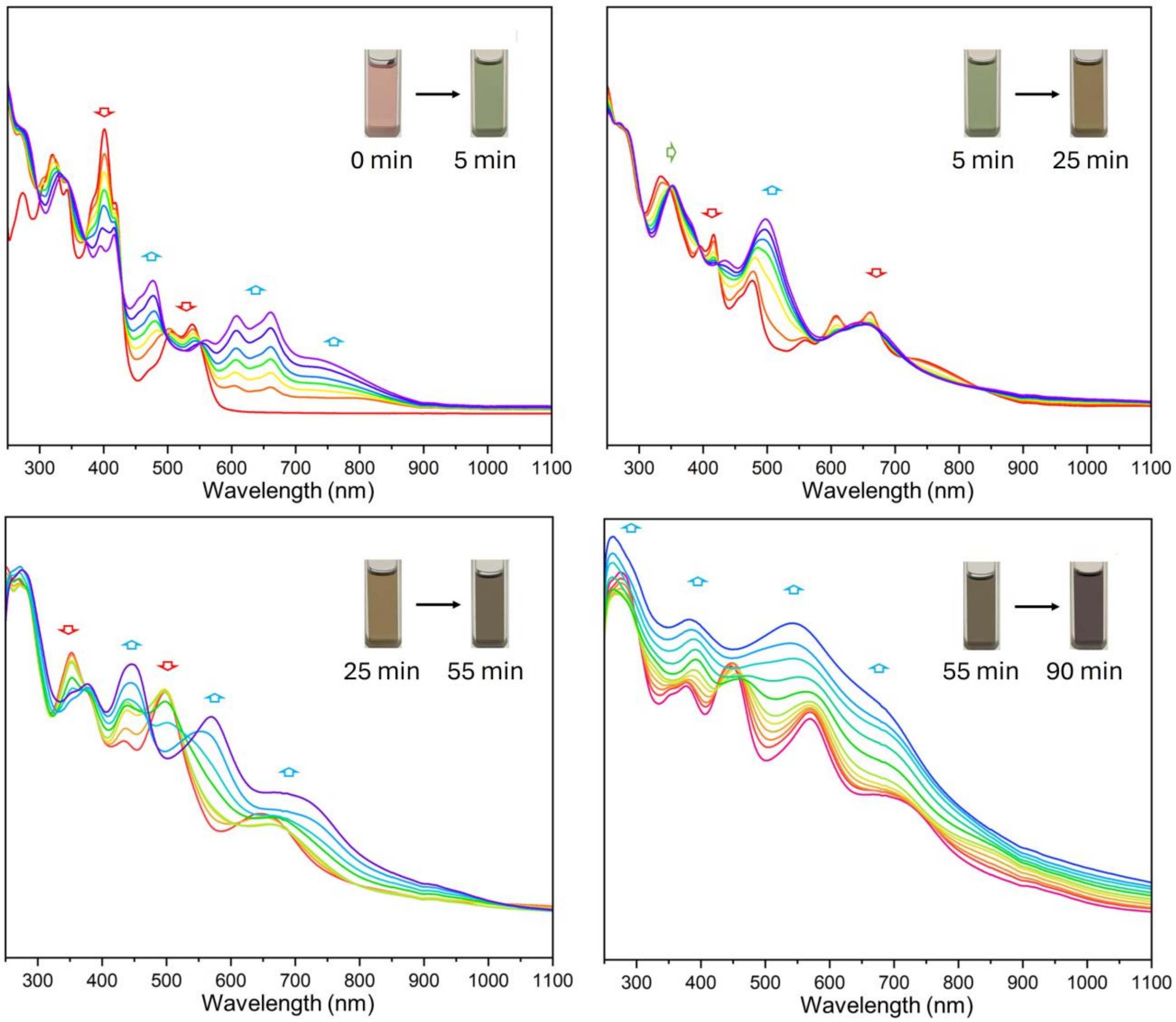

Figure S1. UV-vis-NIR absorption spectra of Rb/**1** in THF at 25 °C.

Sample preparation: DME (2.0 mL) was added to a quartz cuvette containing **1** (0.2 mg, 0.0001 mmol) inside a glovebox. The cuvette was closed tightly with a PTFE cap with a piece of K metal (1.0 mg, 0.0256 mmol) attached to the inner surface, which prevented the initial reaction and allowed control over the reduction process. The capped cuvette was wrapped with Parafilm and removed from the glovebox. The UV-vis-NIR absorption spectra were measured without K metal initially and then monitored with K metal at different reaction times at 25 °C.

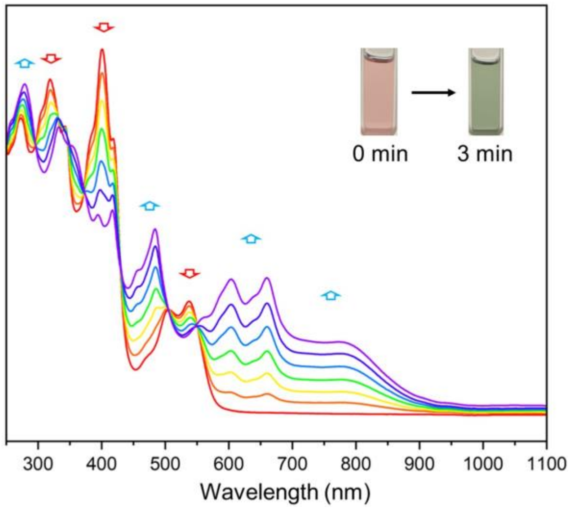

Figure S2. UV-vis-NIR absorption spectra of K/**1** in DME at 25 °C.

Sample preparation: THF (2.0 mL) was added to a quartz cuvette containing **1** (0.2 mg, 0.0001 mmol) and 18-crown-6 (0.1 mg, 0.0004 mmol) inside a glovebox. The cuvette was closed tightly with a PTFE cap with a piece of Rb metal (1.0 mg, 0.0117 mmol) attached to the inner surface, which prevented the initial reaction and allowed control over the reduction process. The capped cuvette was wrapped with Parafilm and removed from the glovebox. The UV-vis-NIR absorption spectra were measured without Rb metal initially and then monitored with Rb metal at different reaction times at 25 °C.

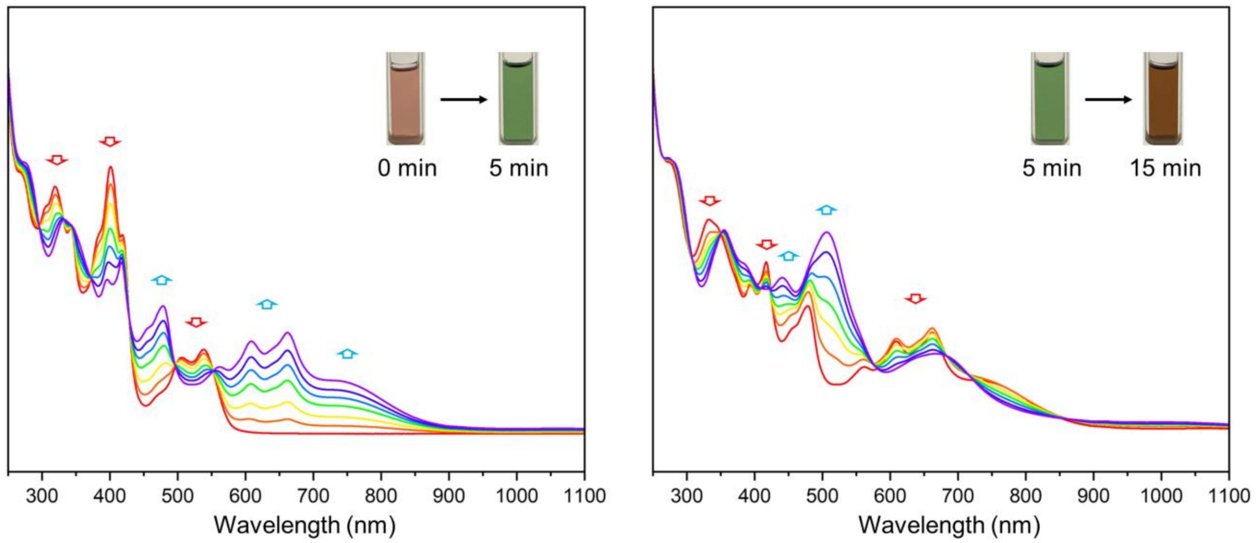

Figure S3. UV-vis-NIR absorption spectra of Rb/**1**/18-crown-6 in THF at 25 °C.

Sample preparation: THF (3.0 mL) was added to a quartz cuvette containing **1** (0.2 mg, 0.0001 mmol) and [2.2.2]cryptand (0.2 mg, 0.0005 mmol) inside a glovebox. The cuvette was closed tightly with a PTFE cap with a piece of Rb metal (1.0 mg, 0.0117 mmol) attached to the inner surface, which prevented the initial reaction and allowed control over the reduction process. The capped cuvette was wrapped with Parafilm and removed from the glovebox. The UV-vis-NIR absorption spectra were initially measured without Rb metal and then monitored with Rb metal at different reaction times at 25 °C.

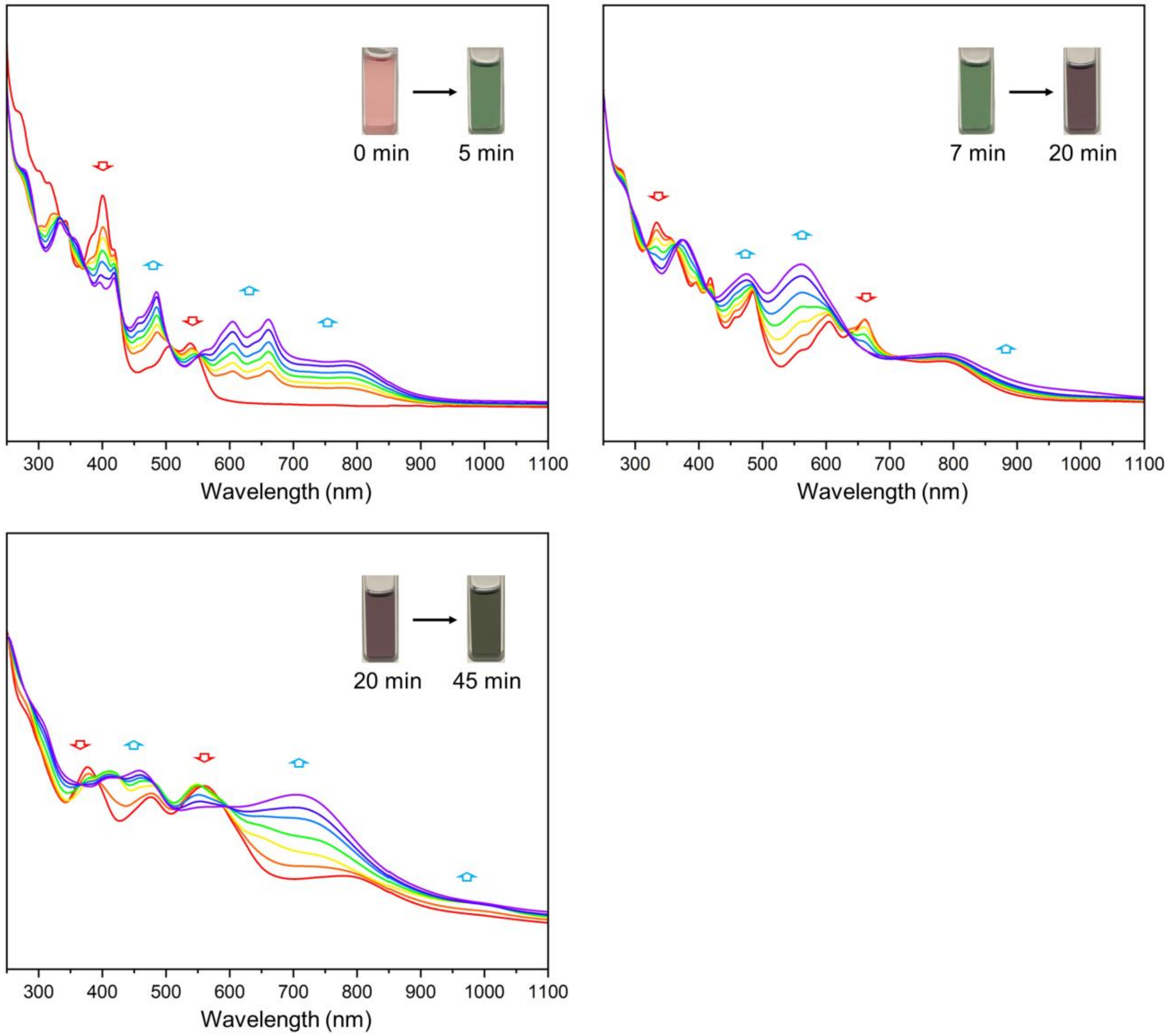

Figure S4. UV-vis-NIR absorption spectra of Rb/**1**/cryptand in THF at 25 °C.

III. NMR Spectroscopic Investigation

Sample preparation: THF-d_8 (0.60 mL) was added to an NMR tube containing excess Rb metal (2.0 mg, 0.023 mmol) and **1** (1.0 mg, 0.0007 mmol). The tube was sealed under argon. The ^{1}H NMR spectra of **1** were collected immediately, and spectra of *in situ* generated anions were collected at different reaction times. After 24 hours, the solution was exposed to dry oxygen by opening the tube and its spectrum was recorded as an O_2 quenched product.

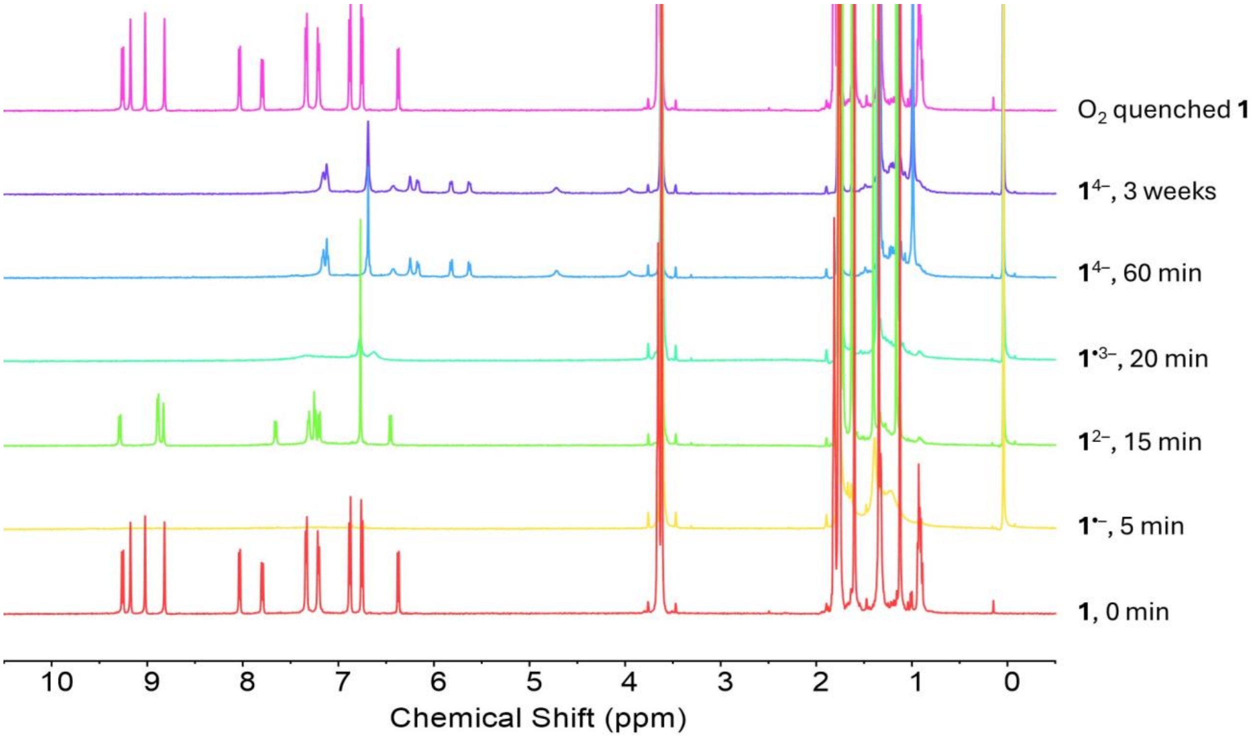

Figure S5. ^{1}H NMR spectra of **1**, *in situ* generated anions, along with the product quenched by O_2 at 25 °C in THF-d_8. Note: The ^{1}H NMR spectrum of $\mathbf{1}^{4-}$ remained the same for three weeks.

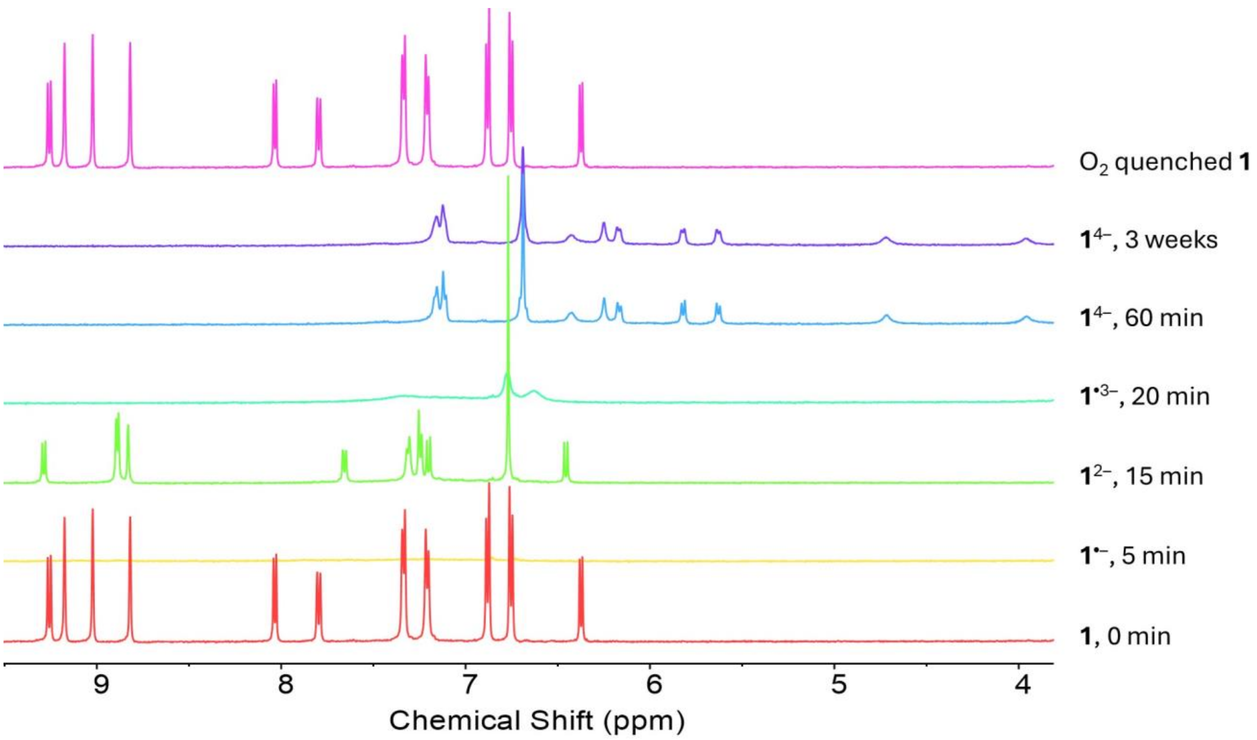

Figure S6. ^{1}H NMR spectra of **1**, *in situ* generated anions, along with the product quenched by O_2 at 25 °C in THF-d_8, aromatic region.

Note: The ^{1}H NMR spectrum of $\mathbf{1}^{4-}$ remained the same for three weeks.

IV. EPR Spectroscopic Investigation

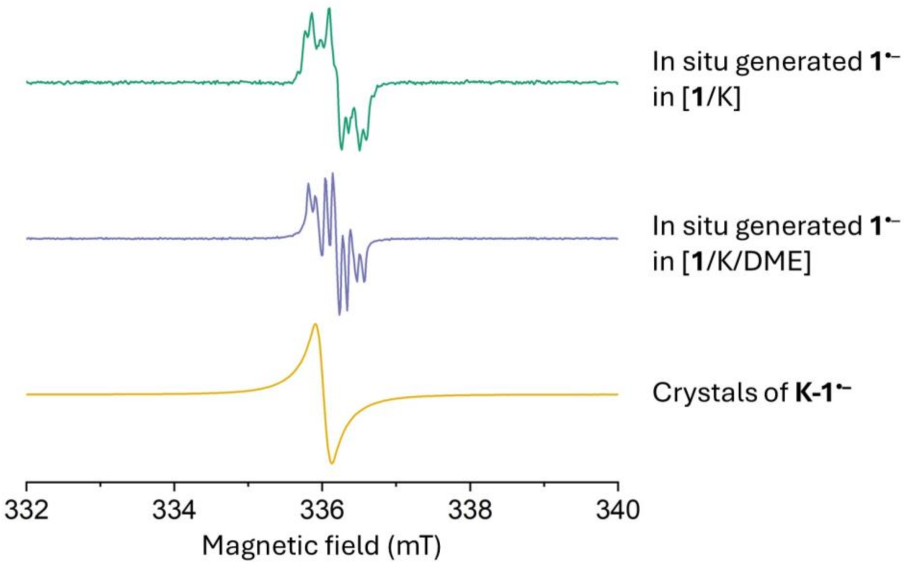

Figure S7. EPR spectra of *in situ* generated monoanion **1**$^{\bullet-}$ under different conditions (solution) and crystals of **K-1**$^{\bullet-}$ (solid) at 30 °C.

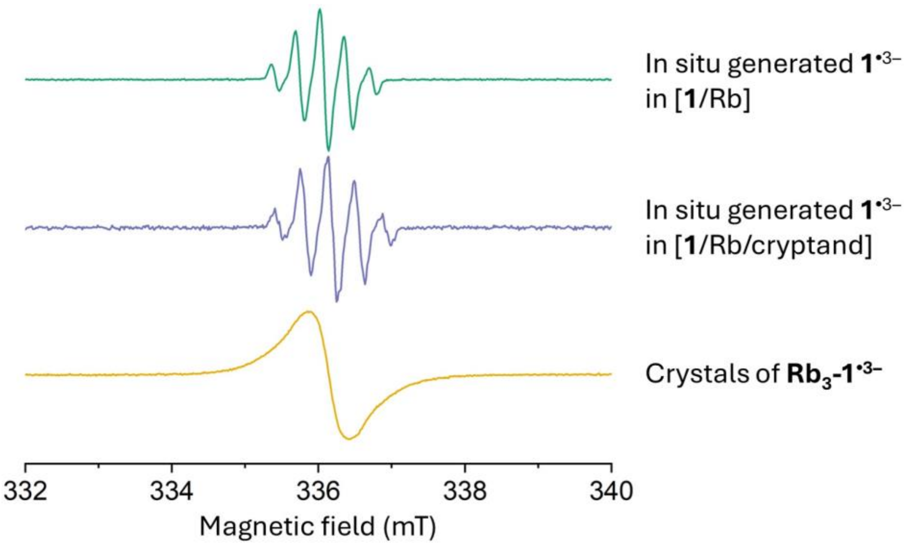

Figure S8. EPR spectra of *in situ* generated trianion **1**$^{\bullet3-}$ under different conditions (solution) and crystals of **Rb$_3$-1**$^{\bullet3-}$ (solid) at 30 °C.

V. Crystal Structure Solution and Refinement

Data collection of **K-1$^{•-}$** and **Rb$_3$-1$^{•3-}$** was performed at 100(2) K on a Huber Kappa system with a DECTRIS PILATUS3 X 2M(CdTe) pixel array detector using ϕ scans (synchrotron radiation at λ = 0.41328 Å) located at the Advanced Photon Source, Argonne National Laboratory (NSF's ChemMatCARS, Sector 15, Beamline 15-ID-D). The dataset's reduction and integration were performed with the Bruker software package SAINT (version 8.38A).[3] Data were corrected for absorption effects using the empirical methods as implemented in SADABS (version 2016/2).[4] Data collection of **Rb$_2$-1^{2-}** was performed at 100.00(10) K on a Rigaku XtaLAB Synergy-S X-ray diffractometer equipped with a HyPix-6000HE hybrid photon counting (HPC) detector and a microfocus Cu-Kα radiation (λ = 1.54178 Å). Data collection strategy to ensure completeness and desired redundancy were determined using CrysAlis^{Pro}.[5] Data processing was performed using CrysAlis^{Pro}.[5] Empirical absorption correction was applied using the SCALE3 ABSPACK scaling algorithm.[6] The structures were solved by SHELXT (version 2018/2)[7] and refined by full-matrix least-squares procedures using the Bruker SHELXTL (version 2019/2)[8] software package through the OLEX2 graphical interface.[9] All non-hydrogen atoms, including those in disordered parts, were refined anisotropically. Hydrogen atoms were included in idealized positions for structure factor calculations with U_{iso}(H) = 1.2 U_{eq}(C) and U_{iso}(H) = 1.5 U_{eq}(C) for methyl groups. In the structure model, two *tert*-butylphenyl groups, one *tert*-butyl group, and all dimethoxyethane molecules were found to be disordered. All disordered molecules and groups were modeled with two orientations with their relative occupancies refined. The geometries of the disordered parts were restrained to be similar. The anisotropic displacement parameters of the disordered molecules in the direction of the bonds were restrained to be equal with a standard uncertainty of 0.004 Å^2. They were also restrained to have the same U_{ij} components, with a standard uncertainty of 0.01 Å^2. In each unit cell of **K-1$^{•-}$**, two hexanes solvent molecules were found to be severely disordered and removed by the Olex2's solvent mask subroutine.[9] The total void volume was 1,959.6 Å^3, equivalent to 16.61 % of the unit cell's total volume. Further crystal and data collection details are listed in Table S1.

Table S1. Crystallographic data of **K-1$^{\bullet-}$**, **Rb$_2$-1^{2-}**, and **Rb$_3$-1$^{\bullet 3-}$**.

Compound	**K-1$^{\bullet-}$**	**Rb$_2$-1^{2-}**	**Rb$_3$-1$^{\bullet 3-}$**
Empirical formula	$C_{126}H_{160}KO_6$	$C_{136}H_{170}Rb_2O_{14.5}$	$C_{186}H_{270}N_6Rb_3O_{25.5}$
Formula weight	1809.63	2207.65	3254.47
Temperature (K)	100(2)	100(2)	100(2)
Wavelength (Å)	0.41328	1.54178	0.41329
Crystal system	Orthorhombic	Tetragonal	Triclinic
Space group	$Cmc2_1$	$I4_1/a$	P–1
a (Å)	26.623(2)	27.49950(10)	14.192(2)
b (Å)	12.2818(9)	27.49950(10)	23.883(4)
c (Å)	36.081(3)	70.8136(5)	28.136(5)
α (°)	90.00	90.00	88.137(2)
β (°)	90.00	90.00	80.210(3)
γ (°)	90.00	90.00	77.124(2)
V (Å^3)	11797.7(16)	53550.8(5)	9161(3)
Z	4	16	2
ρ_{calcd} (g·cm^{-3})	1.019	1.095	1.180
μ (mm^{-1})	0.035	1.385	0.218
F(000)	3932	18816	3486
Crystal size (mm)	0.02×0.04×0.09	0.03×0.09×0.16	0.007×0.015×0.018
θ range for data collection (°)	0.889-17.232	2.592-79.714	0.662-14.362
Reflections collected	227228	105590	173823
Independent reflections	18484 [R_{int} = 0.0790]	28218 [R_{int} = 0.0330]	30165 [R_{int} = 0.1303]
Transmission factors (min/max)	0.5430/0.6372	0.89488/1.00000	0.5541/0.7438
Data/restraints/params.	18484/1091/899	28218/2226/1890	30165/3515/2616
$R1$,[a] $wR2$[b] ($I > 2\sigma(I)$)	0.0663, 0.1843	0.0485, 0.1325	0.0918, 0.2525
$R1$,[a] $wR2$[b] (all data)	0.0732, 0.1943	0.0623, 0.1452	0.1852, 0.3132
Quality-of-fit[c]	1.035	1.031	1.004

$R_{int} = \Sigma|F_o^2 - \langle F_o^2 \rangle| / \Sigma|F_o^2|$

[a] $R1 = \Sigma||F_o|-|F_c|| / \Sigma|F_o|$. [b] $wR2 = [\Sigma[w(F_o^2-F_c^2)^2]/\Sigma[w(F_o^2)^2]]$.

[c] Quality-of-fit = $[\Sigma[w(F_o^2-F_c^2)^2]/(N_{obs}-N_{params})]^{½}$, based on all data.

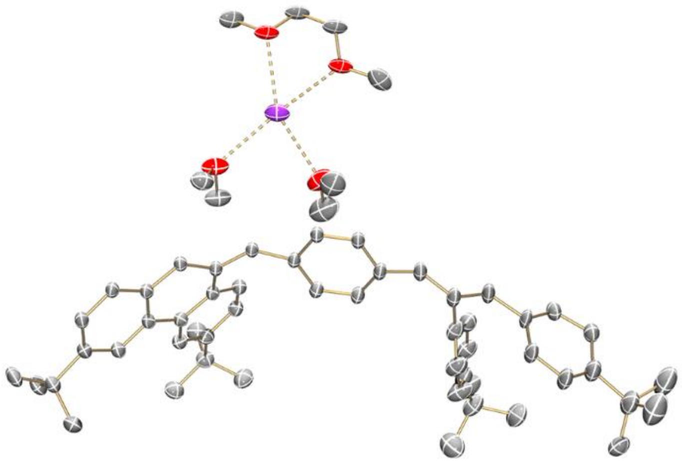

Figure S9. ORTEP drawing of asymmetric unit of **K-1**$^{\bullet-}$, drawn with thermal ellipsoids at the 40% probability level. Hydrogen atoms are omitted for clarity. Color key: C gray, O red, K dark orchid.

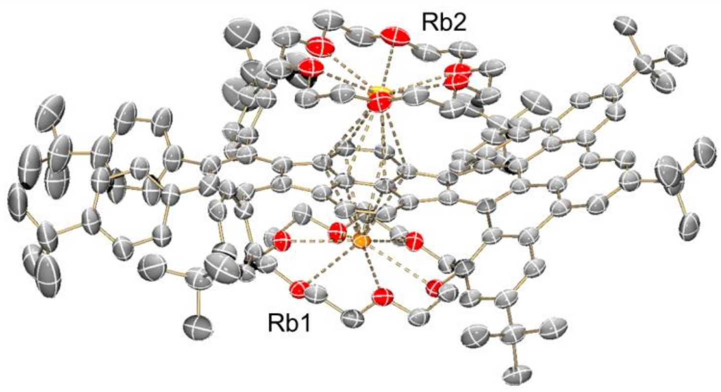

Figure S10. ORTEP drawing of asymmetric unit of **Rb$_2$-1**$^{2-}$, drawn with thermal ellipsoids at the 40% probability level. Hydrogen atoms are omitted for clarity. Color key: C gray, O red, Rb orange.

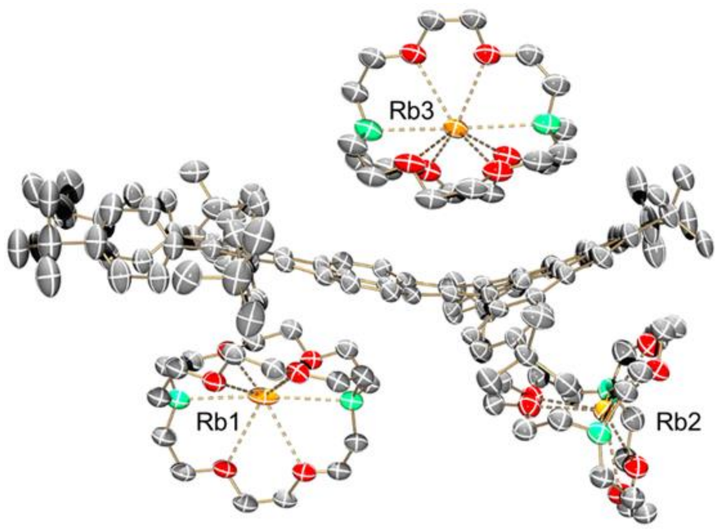

Figure S11. ORTEP drawing of asymmetric unit of **Rb_3-$1^{•3-}$**, drawn with thermal ellipsoids at the 40% probability level. Hydrogen atoms are omitted for clarity. Color key: C gray, O red, N spring green, Rb orange.

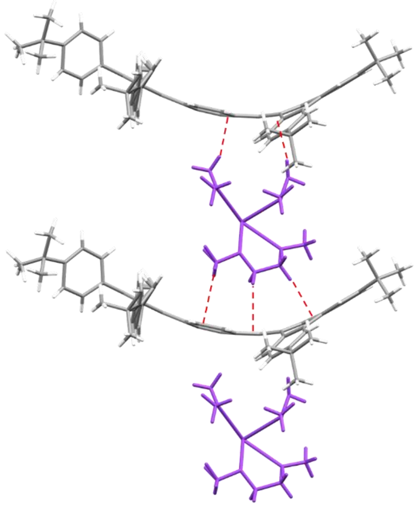

Figure S12. C–H···π interactions (2.419(6)–3.059(6) Å) between the cationic moieties and the **$1^{•-}$** core.

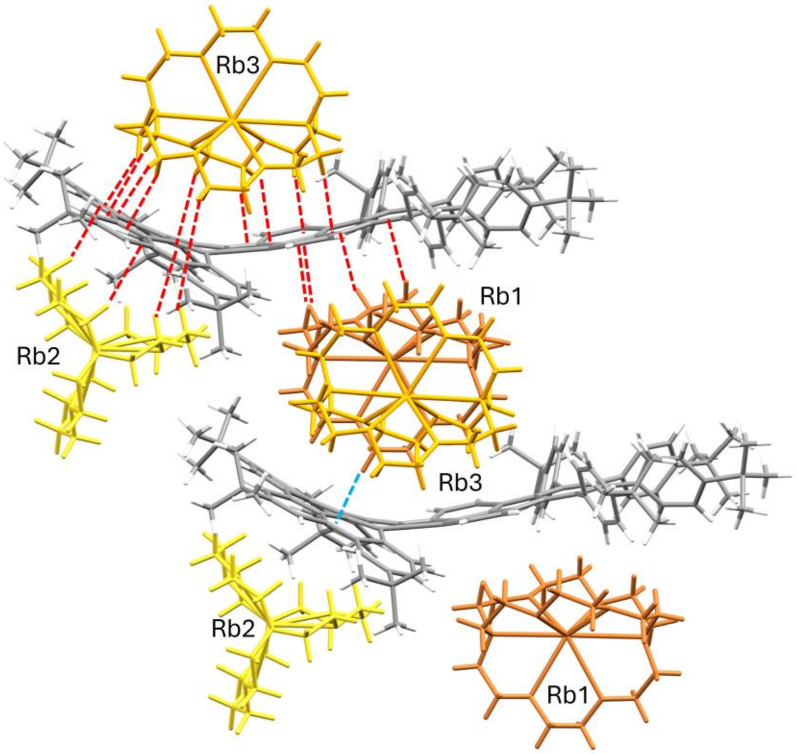

Figure S13. C–H···π interactions (2.456(11)–3.062(11) Å) between the cationic moieties and the $\mathbf{1}^{\bullet 3-}$ core. The intramolecular C–H···π interactions are shown in red and the intermolecular C–H···π interaction (2.534(11) Å) is shown in blue. Independent cations are highlighted in different shades of orange.

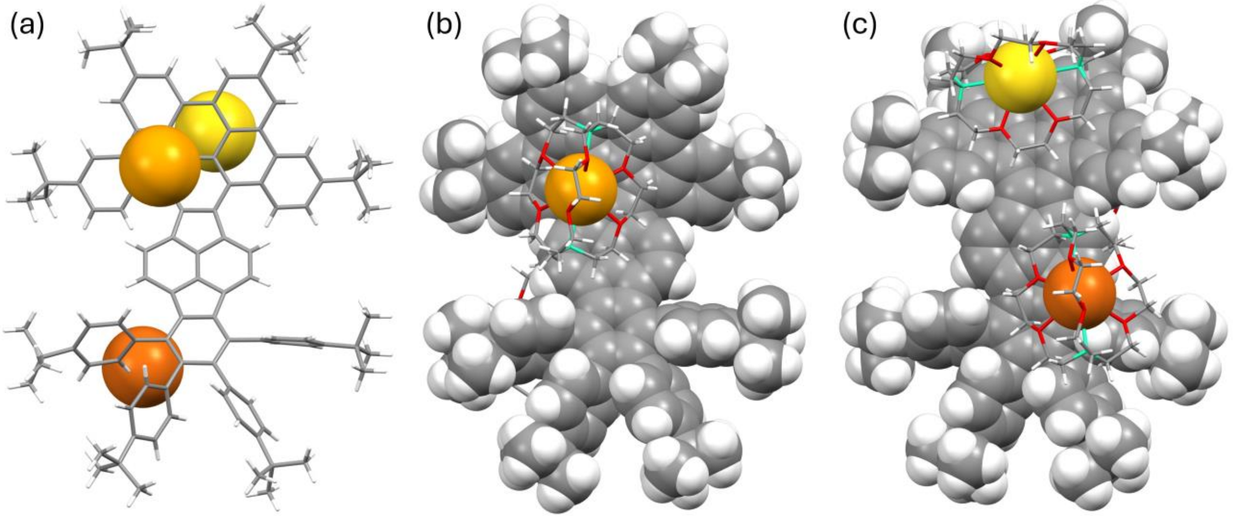

Figure S14. The relative positions of Rb^+ moieties to the $\mathbf{1}^{\bullet 3-}$ core, (a)-(b) top view and (c) bottom view.

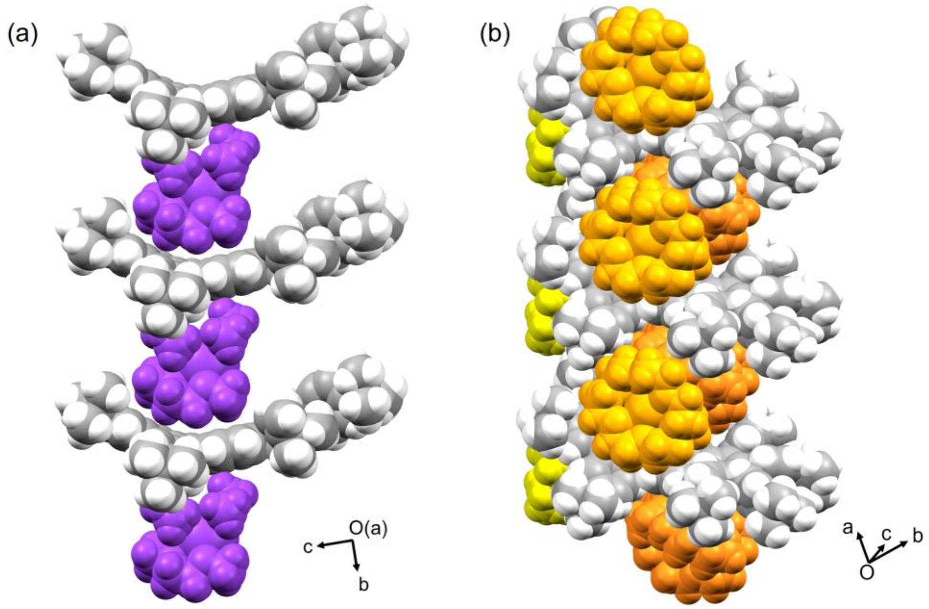

Figure S15. 1D columns of (a) **K-1$^{•–}$** and (b) **Rb$_3$-1$^{•3–}$**, space-filling models. The cationic Rb$^+$ moieties are shown in different shades of orange.

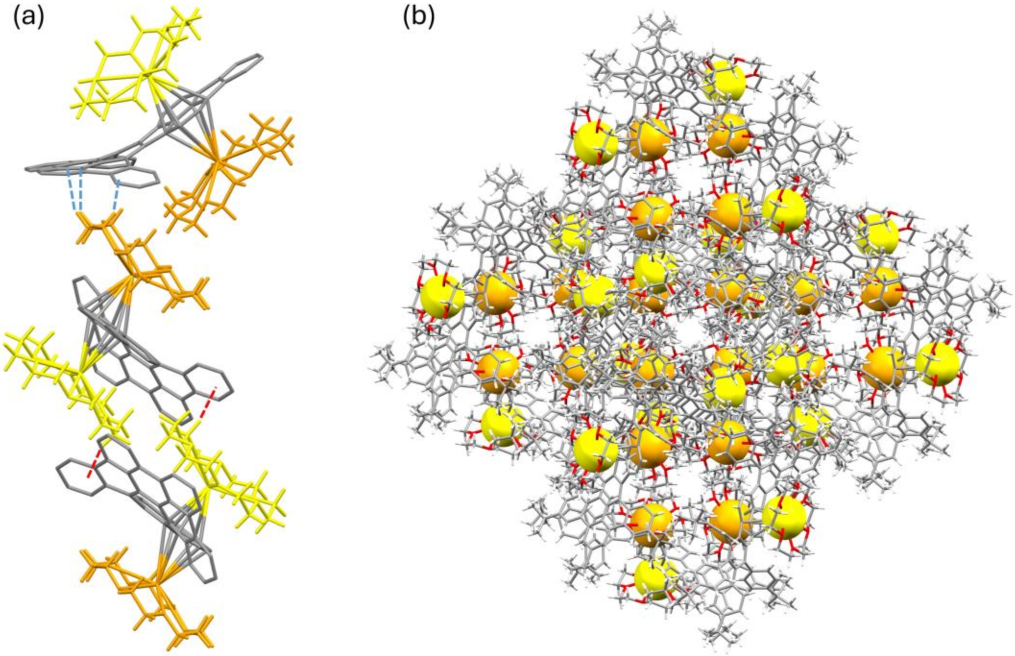

Figure S16. (a) C–H···π interactions between the cationic moieties and the **1**$^{2–}$ core in **Rb$_2$-1$^{2–}$**, capped-stick model. The phenyl substituents, *t*Bu groups, and hydrogen atoms on the anion are omitted for clarity. The C–H···π interactions within the tetrahedral subunit **[Rb$_2$-1$^{2–}$]$_4$** are shown in blue, the C–H···π interaction that builds the 3D network is shown in red. (b) 3D network in **Rb$_2$-1$^{2–}$**, mixed model. Independent Rb cations are highlighted in different shades of orange.

Table S2. Selected C–C bond distances (Å) in **1**, $\mathbf{1}^{\bullet-}$, $\mathbf{1}^{2-}$, and $\mathbf{1}^{\bullet 3-}$, along with a labeling scheme.

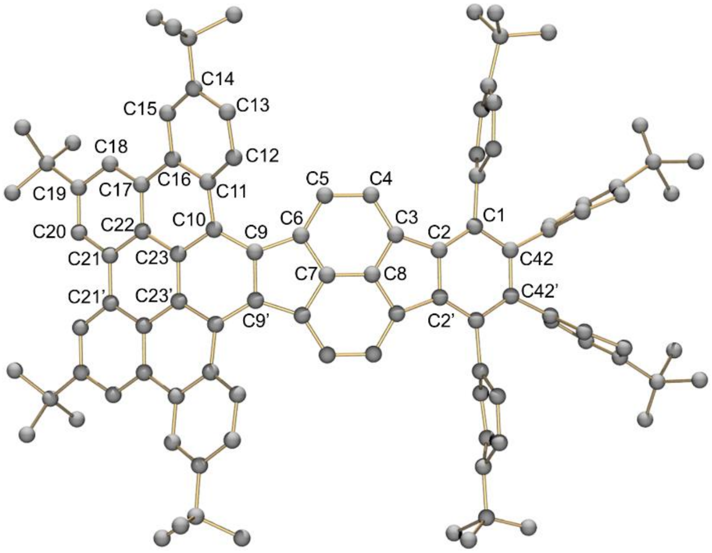

	**1**	$\mathbf{1}^{\bullet-}$	$\mathbf{1}^{2-}$	$\mathbf{1}^{\bullet 3-}$
C1–C2	1.390(3)	1.401(3)	1.409(3)	1.403(9)
C1–C42	1.410(4)	1.413(3)	1.396(3)	1.400(9)
C42–C42’	1.409(3)	1.408(6)	1.420(3)	1.458(9)
C2–C2’	1.429(3)	1.433(5)	1.467(3)	1.481(9)
C2–C3	1.485(3)	1.471(3)	1.436(3)	1.430(9)
C3–C4	1.386(3)	1.408(3)	1.438(3)	1.447(9)
C3–C8	1.409(3)	1.415(3)	1.411(3)	1.433(9)
C4–C5	1.427(4)	1.406(3)	1.382(3)	1.369(9)
C10–C11	1.465(3)	1.457(3)	1.459(3)	1.459(9)
C11–C12	1.402(4)	1.414(3)	1.408(3)	1.403(10)
C11–C16	1.407(4)	1.408(3)	1.410(3)	1.431(9)
C12–C13	1.373(4)	1.375(3)	1.377(3)	1.387(9)
C13–C14	1.399(4)	1.413(3)	1.406(3)	1.390(10)
C14–C15	1.379(4)	1.384(3)	1.376(3)	1.398(9)
C15–C16	1.405(3)	1.409(3)	1.411(3)	1.414(9)
C16–C17	1.470(4)	1.459(3)	1.464(3)	1.423(10)
C9–C10	1.408(3)	1.416(3)	1.423(3)	1.437(9)
C10–C23	1.417(3)	1.415(2)	1.414(3)	1.415(9)

C23–C23’	1.424(3)	1.430(4)	1.428(3)	1.435(8)
C9–C9’	1.433(3)	1.450(4)	1.480(3)	1.489(8)
C6–C9	1.491(3)	1.467(3)	1.439(3)	1.427(9)
C5–C6	1.396(3)	1.417(3)	1.447(3)	1.467(9)
C6–C7	1.410(3)	1.417(3)	1.412(3)	1.418(8)
C7–C8	1.356(4)	1.374(4)	1.385(3)	1.369(9)
C22–C17	1.420(3)	1.424(3)	1.423(3)	1.428(9)
C17–C18	1.394(4)	1.395(3)	1.402(3)	1.482(11)
C18–C19	1.387(4)	1.404(3)	1.380(3)	1.398(11)
C19–C20	1.386(4)	1.390(3)	1.395(3)	1.412(10)
C20–C21	1.395(4)	1.404(3)	1.400(3)	1.436(10)
C21–C22	1.414(4)	1.421(3)	1.423(3)	1.433(9)
C22–C23	1.445(3)	1.438(2)	1.443(3)	1.441(9)
C21–C21’	1.473(3)	1.464(4)	1.459(3)	1.425(9)

* The C–C bonds were shown in red/blue when the distances are shorter/longer compared to the distances in **1**. The bond distances are averaged according to C_s symmetry of the molecule.

Table S3. Selected distances (Å) and angles (°) in **1**, $\mathbf{1}^{\bullet-}$, $\mathbf{1}^{2-}$ and $\mathbf{1}^{\bullet3-}$, along with a labelling scheme.

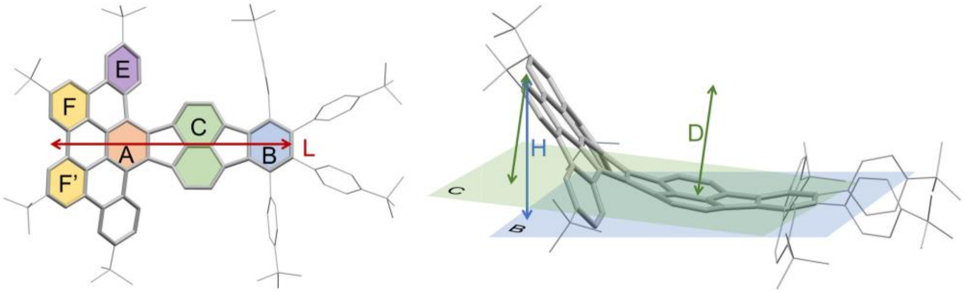

	**1**	$\mathbf{1}^{\bullet-}$	$\mathbf{1}^{2-}$	$\mathbf{1}^{\bullet3-}$
**L**	13.707(7)	13.250(6)	13.885(3)	13.817(9)
**H**	3.447(7)	6.037(6)	1.362(3)	3.104(9)
**D**	3.245(7)	3.811(6)	2.777(3)	3.078(9)
∠**A/B**	24.8(3)	40.6(2)	10.6(2)	21.4(6)
∠**A/C**	23.3(3)	26.7(2)	18.1(2)	21.2(6)
∠**B/C**	2.1(3)	14.0(2)	–7.5(2)*	0.2(6)
Helical angle ∠**A/E**	45.4(3)/46.3(3)	50.6(2)	25.8(2)/26.8(2)	39.3(6)/44.0(6)
Twist angle ∠**F/F'**	1.5(3)	2.3(2)	6.5(2)	8.8(6)

* The negative value shows that ring B bends away from the bowl.

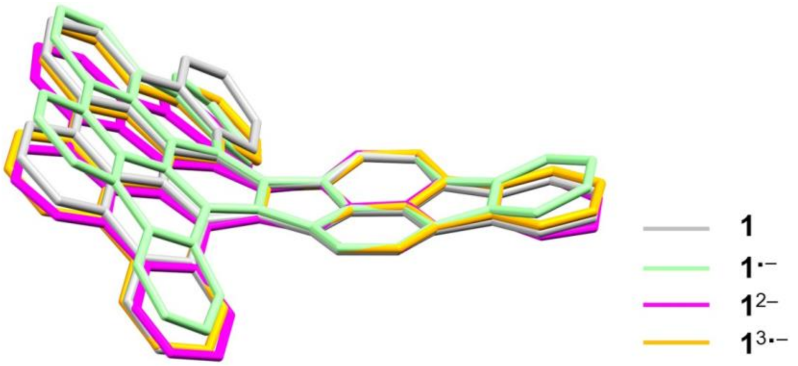

Figure S17 Core deformation in **1**, $\mathbf{1}^{\bullet-}$, $\mathbf{1}^{2-}$, and $\mathbf{1}^{\bullet3-}$. The ^{t}Bu-groups and phenyl rings are omitted for clarity.

Table S4. Planarity measurement for selected rings in **1**, **1**$^{\bullet-}$, **1**$^{2-}$, and **1**$^{\bullet3-}$, along with a labeling scheme.

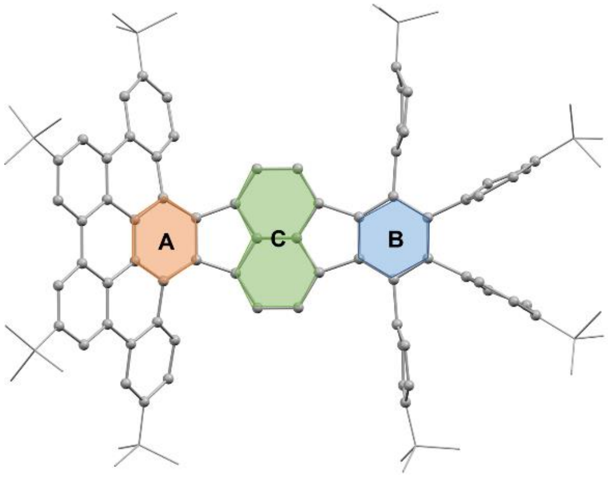

	**A**	**B**	**C**
**1**	–0.003x–0.053y+0.999z+10.645=0 RMSD/A: 0.079	–0.062x–0.366y–0.928z–17.266=0 RMSD/A: 0.004	0.035x+0.344y+0.938z+17.006=0 RMSD/A: 0.078
**1**$^{\bullet-}$	0.972y+0.235z+11.412= 0 RMSD/A: 0.075	0.891y–0.454z–1.281=0 RMSD/A: 0.010	0.974y–0.226z+2.622=0 RMSD/A: 0.063
**1**$^{2-}$	–0.258x+0.715y–0.649z–9.295=0 RMSD/A: 0.078	–0.115x+0.808y–0.578z–6.455=0 RMSD/A: 0.021	–0.016x+0.860y–0.510z–2.957=0 RMSD/A: 0.050
**1**$^{\bullet3-}$	–0.639x–0.355y+0.682z–7.260=0 RMSD/A: 0.077	0.800x+0.030y–0.599z+5.856=0 RMSD/A: 0.009	–0.800x–0.034y+0.599z–5.859=0 RMSD/A: 0.069

VI. Computational Studies

Quantum chemical calculations were performed using the ORCA program suite[10] (versions 5.0.4. and 6.0.0). Geometries were optimized at the PBE0/def2-TZVP level of theory.[11,12] In all cases, we adopted the D3-BJ dispersion correction scheme as proposed by Grimme.[13] For efficient evaluation of Coulomb- and exchange integrals, the RIJ-COSX approximation was used.[14,15] The solvent was modeled using the Conductor-like Polarizable Continuum Model[16] (CPCM) with parameters for THF (ε_r = 7.25, n_{frac} = 1.4070, R_{solv} = 1.3 Å).

Hyperfine coupling (HFC) tensors were calculated employing the TPSS exchange-correlation functional[17] and adopting the EPR-III basis set[18] (and still including the CPCM correction). As this basis is not available for K and Rb, the x2c-TZVPall-s basis[19] set was used for K and Rb. Only the most abundant isotopes were considered. EPR spectra were simulated in the isotropic limit using the EPRsim package by Stephan Rein.[20] The center field was set to 336 mT with a sweep width of ± 4 mT. The spectra were broadened adopting a Voigtian line shape with 0.07 mT Gaussian and 0.07 mT Lorentzian line widths (full width at half maximum, FWHM).

HOMA (Harmonic oscillator model of aromaticity) values are defined as[21]

$$HOMA = 1 - \frac{\alpha}{n}\sum_{i}\left(R_{opt} - R_i\right)^2$$

where *n* is the number of bonds in the ring, and *i* numbers the individual bonds. We are using the C–C reference bond length R_{opt} = 1.388 Å and α = 257.7 of Ref. 21.

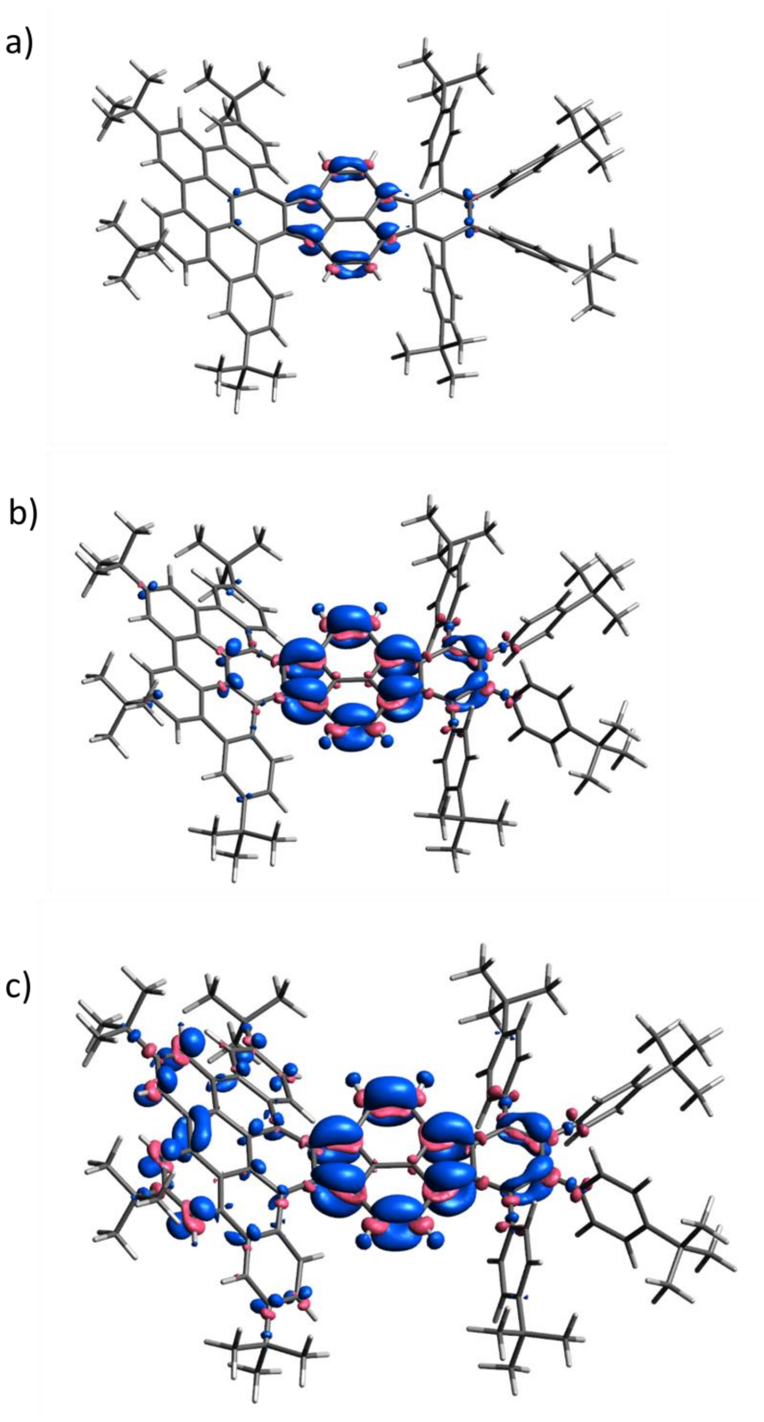

Figure S18. Difference electron density maps of the a) monoanion $\mathbf{1}^{\bullet-}$, b) dianion $\mathbf{1}^{2-}$, and c) trianion $\mathbf{1}^{\bullet3-}$ with respect to the neutral molecule **1** (level: PBE0/def2-TZVP). Lobes are plotted at an isovalue of 0.003 e/bohr^3.

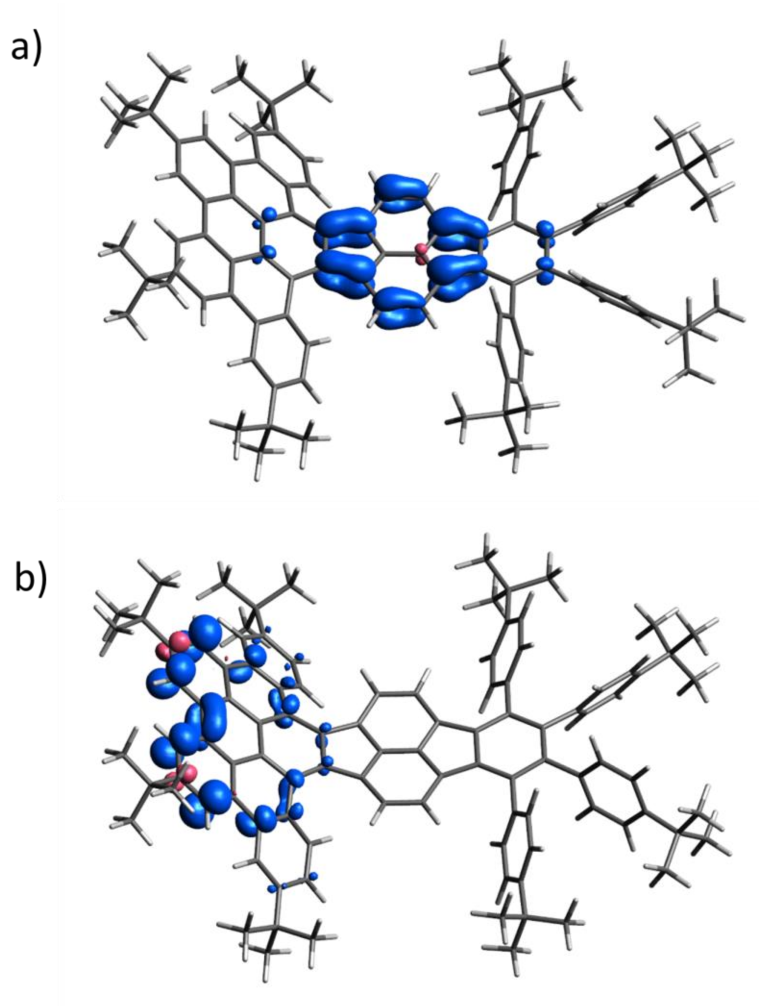

Figure S19. Spin density maps of the a) monoanion $\mathbf{1}^{\bullet-}$, b) trianion $\mathbf{1}^{\bullet 3-}$ (level: PBE0/def2-TZVP). Lobes are plotted at an isovalue of 0.003 e/bohr^3.

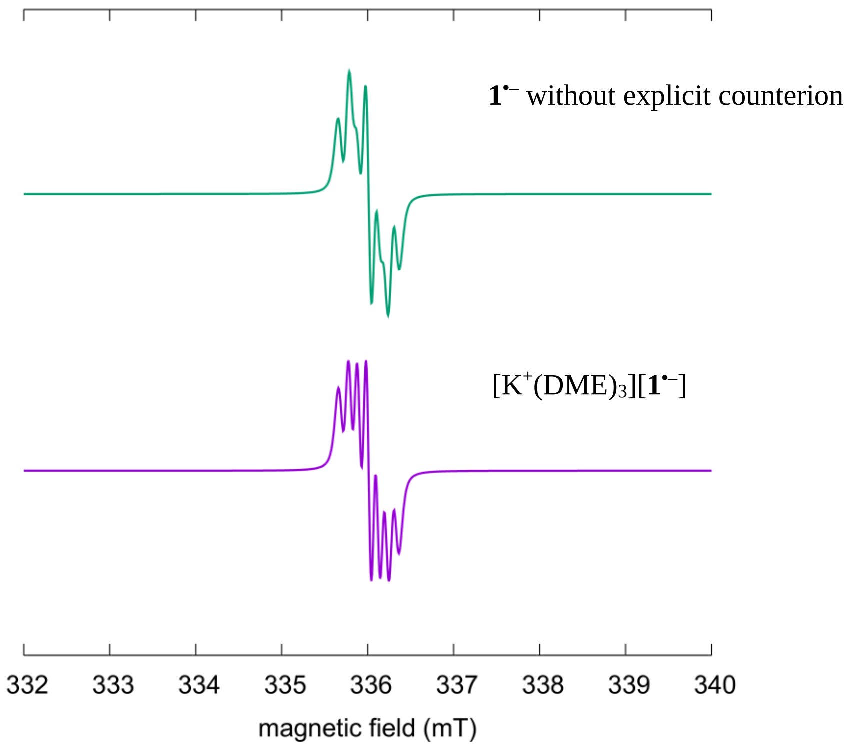

Figure S20. Simulated EPR spectra of the monoanion **1**$^{\bullet-}$ from DFT hyperfine couplings, without (top) and with (bottom) explicit counterion (level: TPSS/EPR-III).

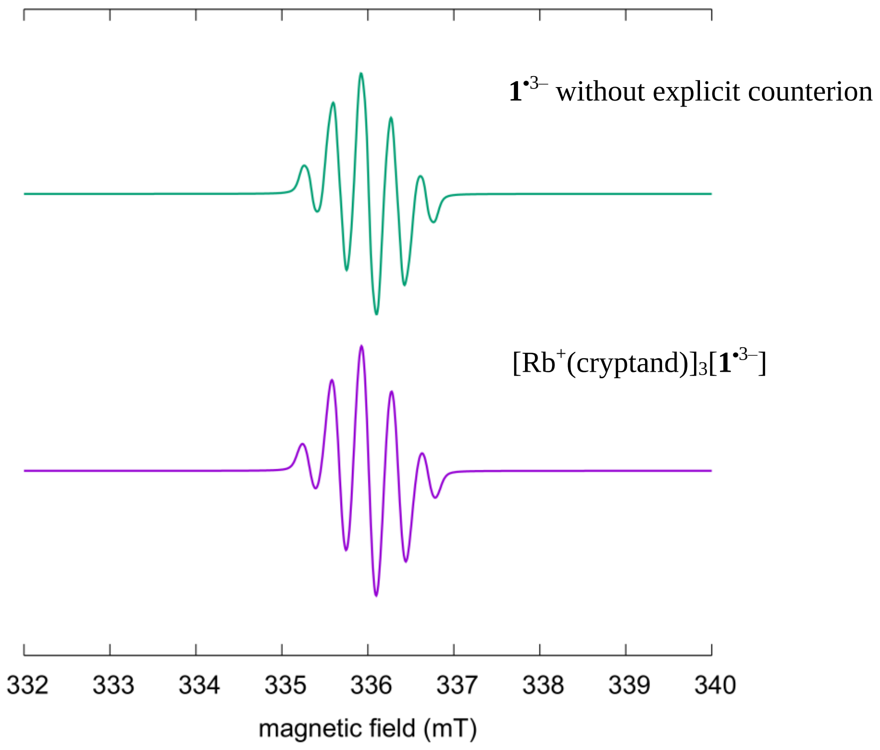

Figure S21. Simulated EPR spectra of the trianion $\mathbf{1}^{\bullet 3-}$ from DFT hyperfine couplings, without (top) and with (bottom) explicit counterions (level: TPSS/EPR-III).

Table S5. Selected C–C distances (Å) in **1**, **1**$^{•–}$, **1**$^{2–}$, and **1**$^{•3–}$ from DFT-optimized structures, along with a labelling scheme.

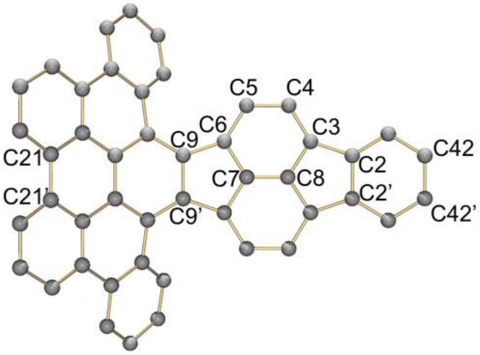

	**1**	**1**$^{•–}$		**1**$^{2–}$		**1**$^{•3–}$	
		w/o counter ions	w counter ions	w/o counter ions	w counter ions	w/o counter ions	w counter ions
C2–C2’	1.426	1.442	1.439	1.464	1.459	1.469	1.464
C2–C3	1.478	1.457	1.455	1.433	1.432	1.432	1.430
C3–C4	1.383	1.407	1.406	1.430	1.429	1.432	1.428
C3–C8	1.403	1.405	1.403	1.410	1.406 / 1.410	1.412	1.409
C4–C5	1.427	1.403	1.402	1.384	1.380 / 1.384	1.386	1.384
C5–C6	1.387	1.415	1.414	1.440	1.437 / 1.440	1.440	1.436
C6–C7	1.403	1.407	1.403	1.411	1.407 / 1.414	1.412	1.410
C7–C8	1.360	1.369	1.367	1.376	1.378	1.375	1.373
C6–C9	1.483	1.457	1.453	1.431	1.425 / 1.436	1.435	1.435 / 1.430
C9–C9’	1.426	1.449	1.448	1.479	1.469	1.461	1.459
C21–C21’	1.456	1.459	1.457	1.460	1.459	1.432	1.430
C42–C42’	1.401	1.411	1.409	1.419	1.422	1.420	1.423

* The ^{t}Bu-groups and σ-phenyl rings are omitted for clarity. The bonds lengths were shown in red/blue when the distances are shorter/longer compared to the distances in **1**.

Table S6. HOMA values from experimental crystal and computed structures.

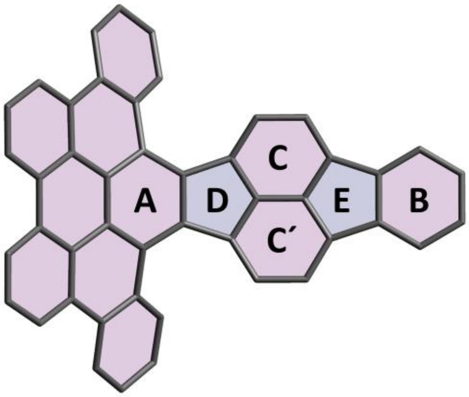

	**1**		$\mathbf{1}^{\bullet-}$			$\mathbf{1}^{2-}$			$\mathbf{1}^{\bullet 3-}$		
ring	Exp.[a]	DFT[b]	Exp.[a]	DFT[b]	DFT[c]	Exp.[a]	DFT[b]	DFT[c]	Exp.[a]	DFT[b]	DFT[c]
**A**	0.76	0.84	0.63	0.72	0.74	0.40	0.48	0.57	0.18	0.50	0.55
**B**	0.86	0.89	0.83	0.83	0.85	0.65	0.68	0.71	0.38	0.65	0.68
**C / C'**	0.85	0.88	0.85	0.90	0.91	0.69	0.76	0.76 / 0.79	0.26 / 0.47	0.75	0.78
**D**	-0.24	-0.03	0.08	0.29	0.36	0.29	0.32	0.42	0.18	0.44	0.49
**E**	-0.10	0.07	0.11	0.33	0.38	0.37	0.44	0.50	0.11	0.41	0.48

* Values computed from: [a] crystal structure, [b] DFT optimized structure without counter ions, [c] DFT optimized structure including counter ions. A HOMA value close to one indicates an aromatic, a value of zero a non-aromatic and significant negative values an antiaromatic character of the respective ring.

Table S7. Absolute isotropic hyperfine coupling parameters $|A_{iso}|$ in MHz computed on the TPSS/EPR-III level. Only values with $|A_{iso}|$ > 1 MHz for least one of the radical species are reported.

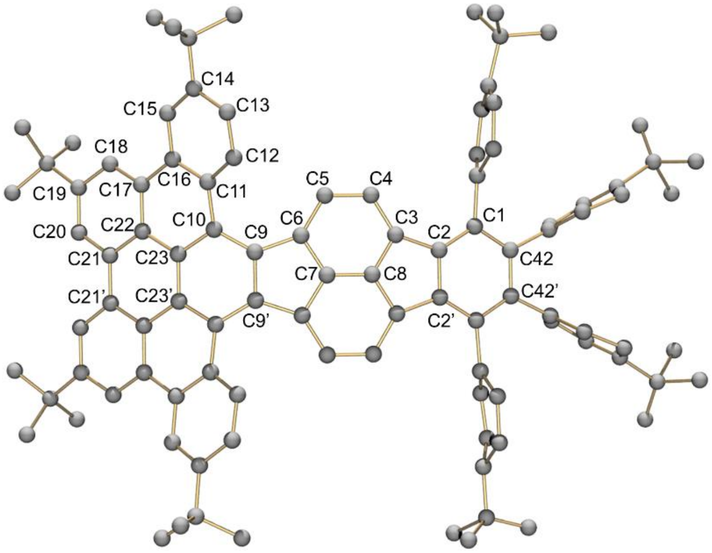

	$\mathbf{1}^{\bullet -}$		$\mathbf{1}^{\bullet 3-}$	
Hydrogen bound to	w/o counter ion	w counter ion	w/o counter ion	w counter ion
C4 / C4’	3.3	3.0 / 3.1	0.2	< 0.1 / 0.2
C5 / C5’	5.5	5.7 / 5.9	0.4	0.3 / 0.4
C13 / C13’	< 0.1	< 0.1	2.0	1.8 / 2.0
C18 / C18’	0.1	0.1	10.3	9.8 / 10.5
C20 / C20’	0.2	0.2	8.4	8.4 / 9.9

VII. References

1. a) N. V. Kozhemyakina, J. Nuss and M. Jansen, *Z. Anorg. Allg. Chem.*, 2009, **635**, 1355; b) W. Stawski, Y. Zhu, Z. Wei, M. A. Petrukhina and H. L. Anderson, *Chem. Sci.*, 2023, **14**, 14109.
2. J. Bergner, C. Walla, F. Rominger, A. Dreuw and M. Kivala, *Chem. Eur. J.*, 2022, **28**, e202201554.
3. SAINT; part of Bruker APEX3 software package (version 2017.3-0): Bruker AXS, 2017.
4. SADABS; part of Bruker APEX3 software package (version 2017.3-0): Bruker AXS, 2017.
5. Rigaku Corporation. Rigaku Oxford Diffraction, CrysAlisPro Software System, Version 1.171.43.92a. 2023.
6. Rigaku Oxford Diffraction. SCALE3 ABSPACK; A Rigaku Oxford Diffraction Program (1.0.11, Gui:1.0.7) (C). 2005.
7. G. M. Sheldrick, *Acta Crystallogr.*, 2015, **A71**, 3.
8. G. M. Sheldrick, *Acta Crystallogr.*, 2015, **C71**, 3.
9. O. V. Dolomanov, L. J. Bourhis, R. J. Gildea, J. A. K. Howard and H. Puschmann, *J. Appl. Crystallogr.*, 2009, **42**, 339.
10. a) F. Neese, *WIREs Comput. Mol. Sci.*, 2012, **2**, 73; b) F. Neese, *WIREs Comput. Mol. Sci.*, 2022, **12**, e1606.
11. C. Adamo and V. Barone, *J. Chem. Phys.*, 1999, **110**, 6158.
12. F. Weigend and R. Ahlrichs, *Phys. Chem. Chem. Phys.*, 2005, **7**, 3297.
13. a) S. Grimme, J. Antony, S. Ehrlich and H. Krieg, *J. Chem. Phys.*, 2020, **132**, 154104; b) S. Grimme, S. Ehrlich and L. Goerigk, *J. Comput. Chem.*, 2011, **32**, 1456.
14. a) F. Neese, F. Wennmohs, A. Hansen and U. Becker, *Chem. Phys.*, 2009, **356**, 98; b) B. Helmich-Paris, B. de Souza, F. Neese and R. Izsák, *J. Chem. Phys.*, 2021, **155**, 104109.
15. a) K. Eichkorn, O. Treutler, H. Öhm, M. Häser and R. Ahlrichs, *Chem. Phys. Lett.*, 1995, **242**, 652; b) F. Weigend, *Phys. Chem. Chem. Phys.*, 2006, **8**, 1057.
16. a) V. Barone and M. Cossi, *J. Phys. Chem. A*, 1998, **102**, 1995; b) M. Garcia-Rates and F. Neese, *J. Comput. Chem.*, 2020, **41**, 922.
17. J. Tao, J. P. Perdew, V. N. Staroverov and G. E. Scuseria, *Phys. Rev. Lett.*, 2003, **91**, 146401.
18. N. Rega, M. Cossi and V. Barone, *J. Chem. Phys.*, 1996, **105**, 11060.
19. Y. J. Franzke, R. Treß, T. M. Pazedera and F. Weigend, *Phys. Chem. Chem. Phys.*, 2019, **21**, 16658.

20. S. Rein EPRsim (version 0.0.4) 2019, available at https://pypi.org/project/EPRsim/
21. T. M. Krygowski, *J. Chem. Inf. Comput. Sci.*, 1993, **33**, 70.
